# Fasting Intervention for Children With Unilateral Renal Tumors to Reduce Toxicity

**DOI:** 10.3389/fped.2022.828615

**Published:** 2022-01-27

**Authors:** Christiaan A. J. Oudmaijer, Winnie M. C. van den Boogaard, Daphne S. J. Komninos, Emma J. Verwaaijen, Hanneke M. van Santen, Marc R. Lilien, Jan H. J. Hoeijmakers, Marc H. W. Wijnen, Marry M. van den Heuvel-Eibrink, Wilbert P. Vermeij

**Affiliations:** ^1^Princess Máxima Center for Pediatric Oncology, Utrecht, Netherlands; ^2^Oncode Institute, Utrecht, Netherlands; ^3^Department of Pediatric Endocrinology, University Medical Center Utrecht, Wilhelmina Childrens Hospital, Utrecht, Netherlands; ^4^Department of Pediatric Nephrology, University Medical Center Utrecht, Wilhelmina Childrens Hospital, Utrecht, Netherlands; ^5^Department of Molecular Genetics, Erasmus MC Cancer Institute, Erasmus University Medical Center, Rotterdam, Netherlands; ^6^Institute for Genome Stability in Aging and Disease, Cologne Excellence Cluster for Cellular Stress Responses in Aging-Associated Diseases (CECAD), University of Cologne, Cologne, Germany

**Keywords:** acute kidney injury, short-term fasting, renal tumor surgery, wilms tumor, toxicity

## Abstract

Childhood renal tumors account for around 6% of all childhood cancers and 90% of these cases are Wilms tumor. In Europe, the SIOP-RTSG approach is considered standard of care and has resulted in five-year survival rates of over 90%. Efforts to decrease toxicity are now being pursued. Short-term fasting (STF), a short but strong reduction in calorie-intake, is associated with improved fitness, enhanced coping with acute physical stress and a lower risk of age-associated diseases. STF temporarily reduces growth to boost resilience, maintenance, and defense-mechanisms, by which toxic side-effects of (oxidative) damage and inflammation are largely prevented. Renal surgery for Wilms tumor carries a risk of acute kidney injury (AKI) and pediatric patients that had an episode of AKI are at increased risk for developing chronic renal disease. STF could mitigate surgery-induced stress and could further improve outcomes. We aim to investigate the effect of STF on renal function recovery after renal tumor surgery by conducting a single-center, prospective, randomized, non-blinded, intervention study. Children diagnosed with a unilateral renal tumor and opting for curative treatment are eligible for inclusion. The main study objective is to investigate the potential decrease in occurrence of AKI due to STF. Secondary objectives include renal function recovery, child's wellbeing, physical functioning, and feasibility of and adherence to STF in children with cancer.

## Introduction

Childhood renal tumors account for around 6% of all childhood cancers (1–3). Most of these cases represent Wilms tumor (WT), also known as nephroblastoma (1–3). About two-thirds of patients with Wilms tumor are diagnosed before the age of 5 years and 95% are diagnosed before the age of 10 years (4), the median age at diagnosis is 43 months for girls and 37 months for boys (5). In Europe, the Renal Tumor Study Group of the International Society of Pediatric Oncology (SIOP-RTSG) guidelines are conducted as best available care and survival is excellent, equal to outcomes from COG-RTG in the USA (3, 6–10). The SIOP-RTSG strategy advocates treatment at dedicated centers and includes preoperative chemotherapy, surgery, and risk-tailored postoperative radiation- and/or chemotherapy (6, 11–15). Preoperative chemotherapy decreases tumor rupture risk during surgery and therefore largely reduces the consequent need for postoperative radiotherapy (16). It downstages the tumor and identifies an extra adverse risk group; the blastemal-type WT, which is considered a high-risk tumor that needs intensified treatment (3, 15). Use of these international regimens, centralization of care, multimodal treatment, response tailored stratification, supportive care strategies and multidisciplinary management have resulted in a significant improvement in survival, with a current overall five-year survival rate approaching 90% (6, 7, 12–15). Therefore, we can also focus on reducing treatment toxicity, which affects long-term quality of life and survival. One of the most striking toxicities after renal tumor treatment is renal function impairment due to sacrificing a substantial percentage of kidney tissue by tumor-nephrectomy, the use of nephrotoxic chemotherapy and, in incidental cases, (high dosage) radiotherapy. Also, a substantial proportion of WT patients carries a germline predisposition which increases not only kidney cancer risk, but also the risk of early kidney failure (17).

An intervention to reduce and/or prevent toxicity is caloric restriction (CR) (18–22). CR, also known as dietary restriction (DR), is defined as a reduced intake of calories, mostly with compensation of vitamins and minerals and without causing malnutrition (23). CR has shown to be associated with extended life span, lower risk of age-associated diseases, improved fitness, and enhanced coping with acute physical stress (18–22). CR can be applied to delay aging or as nutritional preconditioning method before and/or during therapy (18, 22, 24–26). Alternatively, a more stringent and acute method can be used: short-term fasting (STF), which can be performed right before a period of (surgery-induced) stress (22, 27, 28). Recent studies have shown preventive effects of CR and/or STF on aging, genomic stress, ischemia-reperfusion injury (IRI) and acute stress conditions (22, 25, 26, 28–34). Nutritional preconditioning by e.g., CR/STF has been proven feasible and safe in well-nourished patients before living-kidney donation (35–37) and was effective in improving radiological and pathological response during neo-adjuvant chemotherapy treatment for breast cancer (38).

CR and STF redesign energy usage by reducing growth via inhibiting growth hormone (GH) release and decreasing circulating IGF-1, while boosting resilience, maintenance, and defense mechanisms (26, 34, 39–41). Possible explanations for the effect of reduced IGF-1 on longevity are the increased corticosterone levels as a compensatory mechanism and an increase in IGF-BP1, leading to a reduced number of active IGF-1 molecules (42). In addition to reduced GH concentration, the thyrotrophic axis is also downregulated by CR, resulting in decreased concentrations of thyroid stimulating hormone and thyroid hormones (43). During surgery, systemic (IRI-)injury and subsequent formation of reactive oxygen species is unavoidable, leading to elevated cell death and inflammation (44). The protective state induced by CR and STF may reduce the extensiveness of this generalized inflammatory response (31, 33, 34, 39, 45, 46).

Following short-term injury and toxicity experienced by children with cancer, adult survivors of childhood cancer are at risk for long-term nephrotoxicity, i.e., renal dysfunction and high blood pressure (47–51). In limited studies available, an increased risk of severe renal impairment was identified in unilateral WT-survivors (50, 52, 53). In a follow-up study of unilateral WT-survivors, 21% had stage II chronic kidney disease (CKD) (50). In another study in WT-survivors, 53% developed stage II CKD and hypertension was noted in 40% (54). Hence, adult survivors with a solitary kidney have a high relative risk of developing (end-stage) renal disease and this is most likely due to reduced renal mass/eGFR-reserve (55, 56). Pediatric patients that had an episode of acute kidney injury (AKI) are at increased risk for developing chronic renal disease, including hypertension and end-stage renal disease (49, 57, 58). Postoperative AKI is also associated with increased morbidity and longer hospital stay and the magnitude of this risk depends on the severity of AKI (59, 60). STF could potentially reduce hyperfiltration and oxidative stress, specifically on the contralateral kidney, and might therefore lower the risk and extent of acute and late nephrotoxic effects.

To investigate whether STF reduces toxicity and postoperative renal damage, we aim to conduct a single-center, prospective, parallel-group, non-blinded, randomized controlled trial in pediatric patients with renal tumors. These children follow a strictly planned treatment schedule and are in a relatively well condition in comparison with other pediatric oncology patients. This allows implementation of the study-intervention, which can provide further knowledge on the effects of preoperative fasting in pediatric patients. The primary objective of our study is to determine the effect of STF on the occurrence of AKI after tumor-nephrectomy. This study will also further enhance insight into the mechanism and the effect of STF.

## Methods and Analysis

The Fasting Intervention for children with Unilateral Renal Tumors to reduce Toxicity Study (FIURTT-Study) will be conducted at the Princess Máxima Center for Pediatric Oncology, Utrecht, which is the centralized pediatric oncology hospital for all pediatric cancer patients in the Netherlands.

### Study Population

Eligible participants are children with a clinical suspicion of a unilateral malignant renal tumor, without metastatic disease, at the Princess Máxima Center. A diagnosis of a renal tumor, based on MRI, is necessary for participating in the study. Eligible patients will undergo the SIOP-RTSG UMBRELLA regimen (15). Inclusion criteria are a unilateral localized renal tumor for which radical unilateral tumor-nephrectomy is planned and an adequate understanding of the Dutch language. Children will be excluded if they meet any of the following criteria: bilateral renal involvement, low body weight (for subjects younger than 1 year: SD-score < -2 for weight by age, for subjects older than 1 year: SD-score < -2 for weight by height), an underlying metabolic disease, metastatic disease, metachronous disease or no curative treatment possible.

### Intervention

When a child and their caregivers agree to participate, the child will be randomized into a treatment arm. The randomization follows a balanced stratification procedure, per age-group and gender. Subjects randomized to the control arm will follow the normal preoperative guidelines: this includes no intake of food/solids/milk products from 6 h before surgery, but intake of clear liquids (including glucose-rich clear liquids) is allowed until transfer to the preoperative anesthesia care unit. When a subject is randomized to the intervention arm, he/she will undergo the preoperative fasting diet. The duration of STF depends on age: i.e., based on the study from van Veen et al. (61), to avoid unnecessary risk of developing hypoglycaemia. Subjects will be admitted to the hospital 1 day before surgery and therefore STF will be pursued during admission.

The fasting period in the defined age groups are (also shown in figure 1):

- Group 1: 6–24 months (0,5 to 2 years): 10 h of fasting.
- Group 2: 2–7 years: 14 h of fasting.
- Group 3: 7–18 years: 18 h of fasting.

STF is defined as absence of any caloric intake; subjects are free to drink unlimited water, tea, and other clear liquids without calories to maintain a sufficient fluid balance.

### Study Procedures

Subjects will be assessed for blood glucose levels 2 h before the planned time of surgery. If a subject shows any symptoms suggestive of hypoglycaemia (somnolence, trembling, sweating, pale skin color, tachycardia, and/or irritability) during the fasting period, an additional point-of-care blood glucose test will be performed. Escape medication has been defined in the study protocol in the case of hypoglycaemia. For both treatment arms we standardized the induction of anesthesia, maintenance of anesthesia, postoperative pain relief, timing of surgery and IV-fluids for the first 24 h after surgery. General anesthesia will be induced and maintained with Sevoflurane. IV-fluid used during surgery is Kydialite (1% glucose), according to the estimated maintenance requirements following the 4/2/1 rule: 4 cc/kg/hr for the first 10 kg, 2 cc/kg/hr for the second 10 kg, and 1 cc/kg/hr for every kg above 20. It can be supplemented with Ringers lactate at the discretion of the anesthetic team. IV-Fluids during the 24 h after surgery are Ringer's lactate solution or equivalent, according to standard of care protocol (maintaining a diuresis of 1 cc/kg/hr).

Non-invasive interventions that will be conducted during study runtime are questionnaires to analyse aspects of wellbeing and the potential burden of the intervention, wearing an accelerometer and physiotherapeutic assessments. Subjective wellbeing will be analyzed using the PEDSQL-Cancer Module parent form (62, 63). Physiotherapeutic assessments will consist of a short questionnaire to gather information on physical functioning and strength [the SARC-F, (64)], measurements of (skeletal) muscle mass using leg circumference, ultrasonography of quadriceps muscles using a portable linear transducer [Philips Lumify, Philips N.V., Best, The Netherlands (65)] and body composition using bio-electrical Impedance Analysis [Bodystat, QuadScan 4000, Isle of Man, United Kingdom (66)]. Motor function will be evaluated using the Gross Motor Function Measure [GMFM-88 (67)]. Invasive procedures and study appointments will coincide with standard procedures to reduce the burden of participation. Tissue biopsies will be acquired from the removed kidney after diagnostic samples have been taken. Further details and an overview of all procedures are provided in the Appendix III: Study Workflow.

### Primary Outcome

Our primary outcome is AKI on postoperative day 3, 48–72 h after surgery. AKI is defined according to the KDIGO-guidelines (68). The percentage of subjects developing AKI will be measured in both treatment arms. Laboratory measurement of serum creatinine (μmol/L) will be assessed by routine lab procedures and urine output (mL/kg/h) will be calculated by routine clinical procedures. eGFR will be automatically calculated using both the Chronic Kidney Disease Epidemiology Collaboration (CKD-EPI) formula and the Revised Schwartz Equation for Glomerular Filtration Rate (Schwartz-formula).

### Secondary Outcomes

Our secondary endpoints comprise several aspects of the preoperative fasting diet and the postoperative recovery. All the secondary outcomes will be compared between the study arms to identify a possible influence of STF.

- The occurrence of the substages of AKI will be assessed on postoperative day 3. Substages are defined according to KDIGO guidelines (68). Laboratory measurement of creatinine (μmol/L) will be assessed by routine lab procedures and urine output (mL/kg/h) will be calculated by routine clinical procedures.
- Postoperative renal function will be assessed using laboratory measurements, including creatinine (μmol/L), cystatin-C (mg/L), urea (mmol/L), albumin (g/L) and eGFR (ml/min), all assessed by routine lab procedures. These outcomes will be used to determine the postoperative recovery of renal function.
– Renal tissue injury will be investigated on postoperative day 0, 1, 2, 3 and 28. It will be estimated by urine analysis for biomarkers Neutrophil Gelatinase-Associated Lipocalin [NGAL, (69)] and Kidney Injury Molecule-1 [KIM-1, (70)]. These biomarkers will be checked using routine lab procedures at set time points.
- Change in fasting parameters will be calculated using blood samples taken on the day before surgery and on the day of surgery. Laboratory values investigated by routine lab procedures are fasting glucose (mmol/L), fasting insulin (pmolU/L), albumin (g/L), IGF-1 (nmol/L), growth hormone (hGH, mU/L), fT4 (pmol/L), reverse-T3 (nmol/L), fT3 (nmol/L), TSH (mE/L) and β-Hydroxybutyrate (mmol/L).
- Subject wellbeing will be determined via the PedSQL Cancer Module questionnaire (62, 63) and the internally developed behavior questionnaire. Another internally developed questionnaire will be used to investigate the burden of intervention. These questionnaires will be taken at set time points before surgery, at the end of admission and 1 week after surgery.
- Physical activity will be measured via an accelerometer [ActiGraph wgt3x-bt (71)]. Participants will wear the accelerometer in the 7 days before surgery and for another 7 days from postoperative day 4. Open-source software will be used to evaluate physical activity levels during the pre- and postoperative period.
- Physiotherapy assessment will be performed twice. This assessment will evaluate (skeletal) muscle mass [leg circumference, quadriceps muscle ultrasonography (65)], body composition (bio-electrical impedance analysis) and gross motor function (GMFM-88) (67). In children who can walk independently for over 6 months, information on physical functioning and strength will be assessed via SARC-F questionnaire (64).
- Postoperative hospital admission time will be measured in days since surgery.
- Occurrence of peri-operative complications will be carefully assessed. Important complications, such as perioperative tumor spill, hematoma, (wound) infection, intussusception and hypoglycaemia will be investigated and reported in percentages.
- Body weight will be assessed regularly during study runtime at set time points. It will be compared between both study groups and between time points (f.i. pre- and post-fasting).
- Blood pressure will be assessed regularly during study runtime at set study visits and during hospital admittance. We will collect data on blood pressure (mmHg), presence of hypertension (yes/no) and use of anti-hypertensives (yes/no).
- Blood samples and the tissue biopsies obtained will be used to determine upregulation of cytoprotective genes and anti-inflammatory markers associated with the fasting state. Appendix II provides extensive information regarding the histological and molecular analysis. In general, the samples will be used to conduct an expression analysis of genes responsive to fasting, e.g., cytoprotective antioxidant genes, GH/IGF1, and DNA damage and toxicity markers.

### Sample Size Calculation

Data on AKI-occurrence in a cohort of 83 patients, treated at Princess Máxima Center for a renal tumor in the period 2015-2018, was used for our sample size calculation. In this retrospective cohort, AKI after surgery occurred in approximately 37.1% of patients. Power analysis for a one-sample proportion test was applied to compute the number of patients required for this study (72). The statistical program G^*^Power, version 3.1, was used to compute the sample size. To detect a change in proportion from 37 to 18% with 80% power, using a 5%-level two-sided test, 50 patients are needed.

### Recruitment

Inclusion will take place during the 3rd and 4th week of the preoperative chemotherapy period. Eligible subjects will be identified by the principal investigator and/or their treating physician. The coordinating investigator will provide detailed information, both orally and in writing, about the aims, design, and the study procedures. The subject and their caregivers will be offered a reflection period of 1 week until their next outpatient clinic visit, at which the informed consent form will be signed.

### Randomization

The computer program Castor EDC (Amsterdam, the Netherlands), used in compliance with laws and regulations, will be used to randomize study subjects after acquiring informed consent. Randomization will be performed in a 1:1 ratio with stratification for age-group and sex. Due to the nature of our intervention, subjects and investigators cannot be blinded to the result of randomization.

### Data Collection and Management

All study data will be collected from the electronic health system by trained medical personnel. Questionnaires will be completed digitally via an in-house used platform. Each study subject has a personal Case Report Form, of which a blank copy can be supplied on request. If a subject desires to discontinue participation in the trial, we will ask permission to collect measurements conducted according to the standard of care. Retrieved study data will be stored in Castor EDC, in compliance with regulations. The subject-specific study-ID will be linked with the SIOP-RTSG database via their SIOP-ID. A datamanagement plan has been constructed in accordance with local procedure.

### Statistical Analysis

Statistics will be computed using IBM SPSS software version 25.0 (Chicago, IL, USA) or R version 4.0.3 or newer. An intention-to-treat analysis will be conducted to account for the feasibility of the diet. A two-sided significance level of 0.05 will be used for all primary and secondary analyses unless stated otherwise. An analysis will be performed with multiple imputation in the case of missing data. All preoperative / baseline characteristics will be described for the intervention group and the control group. To determine the primary endpoint, we will calculate the proportion of AKI present on postoperative day three after radical nephrectomy in the two treatment arms. Results will be compared by using a Chi-square test for 2 proportions.

In the secondary analysis, a multivariable logistic regression model will be estimated to study the treatment effect of fasting on the occurrence of AKI, adjusted for confounders such as age, sex, weight and compliance to the diet. Additionally, a multinominal logistic regression model will be estimated to investigate the treatment effect of fasting on the substages of AKI. Proportions of substages of AKI will be compared by using the Chi-square test for equality of proportions generalized to more than two independent samples. Due to the presence of repeated measurements, mixed models will be estimated for the following outcomes: postoperative renal function, postoperative renal tissue injury, change in physical activity, quality of life and body weight, to investigate the effect of STF. Confounders such as age, sex, and an interaction term between time and group will be incorporated in each mixed model. Adherence to the diet and occurrence of side effects will be reported as percentage. Change in metabolic, endocrine, and fasting parameters, clinical parameters of mobilization, duration of in-patient hospitalization, occurrence and duration of ICU admission, physiotherapy assessment, occurrence of perioperative complications and upregulation of cytoprotective genes and inflammatory markers will be compared between the two treatment groups by using the *t*-test in the case of continuous outcome variables or non-parametric test in case of violations of normality assumption. The Chi-square test will be used for categorical data.

### Data Monitoring

We classified the risk of our study as negligible and therefore set an independent monitoring visit frequency of once per year, in accordance with the Princess Máxima Center for Pediatric Oncology monitoring plan. Monitoring activities are briefly described as follows; confirming that the Trial Master File and Investigator Site Files are present and complete, confirming that the study staff is adequately instructed on the study procedures, assessment of patient inclusion rate, consent and compliance, source document verification and verification of accessibility of study procedures. The frequency and procedures of auditing will not be changed for our study. Regular auditing, standard for our hospital, will be executed as normal. This process is done independently from the study investigators.

### Potential Harms

(Serious) adverse events will be collected and registered as required by the regulations stated by Princess Máxima Center for Pediatric Oncology, the medical ethical committee, and the Dutch Central Committee on Research Involving Human Subjects (CCMO). Adverse events have been defined as any undesirable experience occurring to a subject during study runtime, whether or not considered related to the intervention. All adverse events reported spontaneously by the subject or observed by the investigator or his/her staff will be recorded, unless these adverse events are part of the normal postoperative time period and are not signs or symptoms of serious postoperative complications (Clavien-Dindo class ≥ 3) (73). A serious adverse event has been defined according to the definition of the CCMO. An elective hospital admission and prolongation of hospitalization after renal tumor surgery will not be considered an adverse event unless it derives from serious postoperative complications (Clavien-Dindo class ≥3) (73).

For this study, no additional invasive procedures are performed, biopsies are acquired from surplus tissue and no additional visits to the hospital are required. Based on previous studies, we do not expect an increase in postoperative complications due to the preoperative fasting intervention. Our STF regimens are based on earlier studies to avoid potential risks (61, 74) and is conducted under supervision. To further reduce the risk of prolonged hypoglycaemia, we will conduct blood glucose tests before surgery and when a subject shows symptoms of hypoglycaemia. Protocols have been constructed for the acute treatment of hypoglycaemia. Combining the low chance of postoperative complications, absence of evidence for an increase in complications due to STF and clear guidelines to avoid hypoglycaemia, we do not expect a higher incidence of complications or any compromises on subject safety. Potential compensation for any participant who suffers harm due to trial participation has been covered by hospital insurance, in accordance with Dutch laws and regulations. Since this preoperative fasting diet is tailored to the age of the subject and the surgery conducted, participants cannot continue this specific fasting diet after completing the study. However, adhering to a fasting diet can be done on an individual basis, if preferred and in consensus with healthcare providers. Knowledge of potential benefits of preoperative fasting diets will be of extensive value for further studies and as a potential treatment strategy for future patients.

## Discussion

With this single-center, prospective, parallel-group, non-blinded randomized controlled trial, we aim to determine the effect of short-term fasting on acute kidney injury, treatment toxicity and postoperative recovery in pediatric patients diagnosed with a Wilms tumor. Our study will provide information on the merits of caloric restriction, in the form of short-term fasting, and its possible future application and possible implementation in pediatric oncology care.

Nutritional preconditioning represents a non-invasive and cost-effective method that could mitigate the effects of acute surgery-induced stress and (postoperative) toxicity. It has previously been associated with health benefits when exerted prolonged against age-related diseases and more acute as preconditioning method against treatment related toxicities, including kidney transplantation surgery and neoadjuvant chemotherapy in adults. It has however, never been applied to children during cancer treatment, who need to cope with treatment-related toxicities during treatment and for decades after successful curation from cancer. A potential drawback observed in some initial adult clinical trials are issues with adherence to the dietary regimen. We have tried to circumvent this issue by including expertise and advise of parents from the Society Childhood Cancer Netherlands (Vereniging Kinderkanker Nederland) into the design of our study. We expect to increase feasibility of our study by, amongst others, scheduling of surgical operations in the morning, thereby having most of the fasting period during the night, making fasting easier, especially for young children. Additionally, smell of and temptations to food are minimized by adjusting room location in the hospital and special door signs indicating a child is fasting. To investigate whether preoperative short-term fasting contributes to a clinically significant reduction in acute kidney injury after radical nephrectomy for a unilateral renal tumor in pediatric patients, without being a significant burden on the pediatric patient, this randomized clinical trial is indicated. It could significantly reduce the extent of treatment-related toxicity, therefore reducing disease burden in the short- and long-term.

Outcomes from this clinical trial would be applicable for renal surgical toxicities throughout the world, reducing treatment toxicities in other facets of pediatric oncology care or toward follow-up studies in multiple other treatment modalities.

## ETHICS AND DISSEMINATION

The medical ethical committee of Utrecht University Medical Center, Utrecht, the Netherlands, has approved the study protocol, patient information files and consent procedures and other study-related documents and procedures. The procedure regarding study amendments has been defined in the study protocol. A substantial amendment is defined as an amendment that is likely to affect to a significant degree: the safety or physical or mental integrity of the subjects of the trial, the scientific value of the trial, the conduct or management of the trial or the quality or safety of any intervention used in the trial. Substantial amendments will be submitted to the medical ethical committee and will only be conducted after approval. Small insubstantial amendments can be made at the principal investigator's discretion and will be notified to the ethical committee. If a protocol amendment has been accepted by the ethical committee, it will be changed accordingly in the trial registry and any previous publications.

Informed consent will be obtained in accordance with relevant laws and regulations (e.g., ICH-GCP) by qualified study researchers or the principal investigator. Because our research will be performed with minors, separate procedures and informed consent files have been constructed for children according to their age and their caregivers. A separate consent has been constructed for collection and use of participant data and biological specimens in future related studies. This consent is not required to participate in the study. Model consent forms have been provided in the appendices.

### Confidentiality

Upon study inclusion, each subject will be assigned a study-ID. All personal or pseudonymized data will be handled with care and in compliance with the EU General Data Protection Regulation. Data will be confidentially saved for a maximum of 15 years after completion of the study, in compliance with Dutch practice and laws. The principal investigator will keep a subject identification log, holding record of the personal data and the subject's study-ID. This record is filed at the investigational site and can only be accessed by the investigator and authorized site staff and data that can be traced to individual persons can only be viewed by authorized personnel. The principal investigator is responsible for final trial data set. Research data can only be viewed by authorized personnel, the research team, members of the healthcare inspection and study monitors. Potential data sharing requests will be approved by a representative of the hospital and the principal investigator.

The principal investigator is responsible for the public disclosure and publication of the research data. Results of the study will be summarized in an article and will be submitted for publication in a scientific journal. Also, all participating subjects or their family will receive a layman's summary of the (final) results of the study, if desired. The trial has also been registered in a Dutch public trial registration registry, available at: http://www.trialregister.nl/trialreg/index.asp). Eligibility for authorship has been defined by the ICMJE guidelines for the role of Authors and Contributors^1^. The full protocol, anonymized dataset and statistical code can be granted after submitting a request to the principal investigator and a representative of the hospital.

## Author Contributions

Specifically, formulating the hypothesis, designing the study, and defining the study-related procedures was performed by CO, WB, JH, MW, MH-E, and WV. CO, WB, DK, JH, MW, MH-E, and WV have written this manuscript. EV, HS, and ML have conducted extensive review. WB, DK, JH, and WV have designed and will conduct the histological and molecular analysis of the samples acquired. During study runtime, the inclusion of potential study subjects will be performed by CO, MW, and MH-E. The collection, management, analysis, and interpretation of data will be performed by CO. The writing of the final report and decision to submit will be made by CO, WB, DK, JH, MW, MH-E, and WV. All authors have been involved extensively in the design phase of this study.

## Funding

The design and implementation of this study was conducted without support of a specific grant from any funding agency in the public, commercial or non-profit sectors. Research and writing of this manuscript were indirectly supported by ONCODE (Dutch Cancer Society). JH was additionally supported by the European Research Council Advanced Grant Dam2Age, NIH grant (PO1 AG017242), the Deutsche Forschungsgemeinschaft - Project-ID 73111208 - SFB 829, WV by the ADPS Longevity Research Award and Regiodeal Foodvalley, and JH and WV by BBoL (NWO-ENW), ZonMW Memorabel (733050810), and EJP-RD TC-NER RD20-113.

**Informed Consent Forms:**

- Patient information letter and Informed Consent form - parents/caretakers.
- Patient information letter and Informed Consent form patients 12-16 years.
- Patient information letter and Informed Consent form patients older than 16 years.

## Conflict of Interest

The authors declare that the research was conducted in the absence of any commercial or financial relationships that could be construed as a potential conflict of interest.

## Publisher's Note

All claims expressed in this article are solely those of the authors and do not necessarily represent those of their affiliated organizations, or those of the publisher, the editors and the reviewers. Any product that may be evaluated in this article, or claim that may be made by its manufacturer, is not guaranteed or endorsed by the publisher.

## Footnotes

^1^ http://www.icmje.org/recommendations/browse/roles-and-responsibilities/defining-the-role-of-authors-and-contributors.html

## Supplementary Material

Supplementary Figure 1 Schematic overview of the FIURTT study.

Supplementary Figure 2 Survival graphic from the fasting test from van Veen et al. (61).

## References

[1] Kalapurakal JA Dome JS Perlman EJ Malogolowkin M Haase GM Grundy P . Management of Wilms' tumour: current practice and future goals. Lancet Oncol. (2004) 5:37–46. 10.1016/S1470-2045(03)01322-6 14700607

[2] Gooskens SL Graf N Furtwängler R Spreafico F Bergeron C Ramírez-Villar GL . Position paper: rationale for the treatment of children with CCSK in the UMBRELLA SIOP-RTSG 2016 protocol. Nat Rev Urol. (2018) 15:309–19. 10.1038/nrurol.2018.14 29485128

[3] Brok J Lopez-Yurda M Tinteren HV Treger TD Furtwängler R Graf N . Relapse of Wilms' tumour and detection methods: a retrospective analysis of the 2001 Renal Tumour Study Group-International Society of Paediatric Oncology Wilms' tumour protocol database. Lancet Oncol. (2018) 19:1072–81. 10.1016/S1470-2045(18)30293-6 29960848

[4] Ries LAG Smith MA Gurney JG Linet M Tamra T Young JL . Cancer Incidence and Survival Among Children and Adolescents: United States SEER Program 1975-1995, SEER Program. Bethesda, MD: National Cancer Institute (1999). p.79.

[5] Breslow N Olshan A Beckwith JB Green DM. Epidemiology of Wilms tumor. Med Pediatr Oncol. (1993) 21:172–81. 10.1002/mpo.2950210305 7680412

[6] D'Angio GJ Breslow N Beckwith JB Evans A Baum H deLorimier A . Treatment of Wilms' tumor. Results of the Third National Wilms' Tumor Study. Cancer. (1989) 64:349–60. 10.1002/1097-0142(19890715)64:2<349::aid-cncr2820640202>3.0.co;2-q 2544249

[7] Metzger ML Dome JS. Current therapy for Wilms' tumor. Oncologist. (2005) 10:815–26. 10.1634/theoncologist.10-10-815 16314292

[8] Vujanić GM Gessler M Ooms A Collini P Coulomb-l'Hermine A D'Hooghe E . The UMBRELLA SIOP-RTSG 2016 Wilms tumour pathology and molecular biology protocol. Nat Rev Urol. (2018) 15:693–701. 10.1038/s41585-018-0100-3 30310143 PMC7136175

[9] Janssens GO Melchior P Mul J Saunders D Bolle S Cameron AL . The SIOP-Renal Tumour Study Group consensus statement on flank target volume delineation for highly conformal radiotherapy. Lancet Child Adolescent Health. (2020) 4:846–52. 10.1016/S2352-4642(20)30183-8 33068550

[10] Mul J van Grotel M Seravalli E Bosman ME van Tinteren H Roy P . Locoregional control using highly conformal flank target volumes and volumetric-modulated arc therapy in pediatric renal tumors: results from the Dutch national cohort. Radiother Oncol. (2021) 159:249–54. 10.1016/j.radonc.2021.04.005 33845042

[11] D'Angio GJ. Pre- or postoperative therapy for Wilms' tumor? J Clin Oncol. (2008) 26:4055–7. 10.1200/JCO.2008.16.5316 18757319

[12] Spreafico F Bellani FF. Wilms' tumor: past, present and (possibly) future. Expert Rev Anticancer Ther. (2006) 6:249–58. 10.1586/14737140.6.2.249 16445377

[13] Tournade MF Com-Nougué C de Kraker J Ludwig R Rey A Burgers JM . Optimal duration of preoperative therapy in unilateral and nonmetastatic Wilms' tumor in children older than 6 months: results of the Ninth International Society of Pediatric Oncology Wilms' Tumor Trial and Study. J Clin Oncol. (2001) 19:488–500. 10.1200/JCO.2001.19.2.488 11208843

[14] Koshinaga T Takimoto T Oue T Okita H Tanaka Y Nozaki M . Outcome of renal tumors registered in Japan Wilms Tumor Study-2 (JWiTS-2): a report from the Japan Children's Cancer Group (JCCG). Pediatr Blood Cancer. (2018) 65:e27056. 10.1002/pbc.27056 29630767

[15] van den Heuvel-Eibrink MM Hol JA Pritchard-Jones K van Tinteren H Furtwängler R Verschuur AC . Position paper: Rationale for the treatment of Wilms tumour in the UMBRELLA SIOP-RTSG 2016 protocol. Nat Rev Urol. (2017) 14:743–52. 10.1038/nrurol.2017.163 29089605

[16] Mitchell C Pritchard-Jones K Shannon R Hutton C Stevens S Machin D . Immediate nephrectomy versus preoperative chemotherapy in the management of non-metastatic Wilms' tumour: results of a randomised trial (UKW3) by the UK Children's Cancer Study Group. Eur J Cancer. (2006) 42:2554–62. 10.1016/j.ejca.2006.05.026 16904312

[17] Segers H Kersseboom R Alders M Pieters R Wagner A van den Heuvel-Eibrink MM. Frequency of WT1 and 11p15 constitutional aberrations and phenotypic correlation in childhood Wilms tumour patients. Eur J Cancer. (2012) 48:3249–56. 10.1016/j.ejca.2012.06.008 22796116

[18] Colman RJ Anderson RM Johnson SC Kastman EK Kosmatka KJ Beasley TM . Caloric restriction delays disease onset and mortality in rhesus monkeys. Science. (2009) 325:201–4. 10.1126/science.1173635 19590001 PMC2812811

[19] Levolger S Engel Svd Wiel-Ambagtsheer GAd IJzermans JNM Bruin RWFd. Caloric restriction is associated with preservation of muscle strength in experimental cancer cachexia. Aging. (2018) 10:4213–23. 10.18632/aging.101724 30591621 PMC6326673

[20] Nakagawa S Lagisz M Hector KL Spencer HG. Comparative and meta-analytic insights into life extension via dietary restriction. Aging Cell. (2012) 11:401–9. 10.1111/j.1474-9726.2012.00798.x 22268691

[21] Robertson LT Mitchell JR. Benefits of short-term dietary restriction in mammals. Exp Gerontol. (2013) 48:1043–8. 10.1016/j.exger.2013.01.009 23376627 PMC3745522

[22] Boogaard WMCvd Heuvel-Eibrink MMvd Hoeijmakers JHJ Vermeij WP. Nutritional preconditioning in cancer treatment in relation to DNA damage and aging. Ann Rev Canc Biol. (2021) 5:161–79. 10.1146/annurev-cancerbio-060820-090737 PMC9037985 35474917

[23] Speakman JR Mitchell SE. Caloric restriction. Mol Aspects Med. (2011) 32:159–221. 10.1016/j.mam.2011.07.001 21840335

[24] Fontana L Partridge L Longo VD. Extending healthy life span–from yeast to humans. Science. (2010) 328:321–6. 10.1126/science.1172539 20395504 PMC3607354

[25] Dorling JL Das SK Racette SB Apolzan JW Zhang D Pieper CF . Changes in body weight, adherence, and appetite during 2 years of calorie restriction: the CALERIE 2 randomized clinical trial. Eur J Clin Nutr. (2020). 10.1038/s41430-020-0593-8 32144378 PMC7415503

[26] Vermeij WP Dollé M Reiling E Jaarsma D Payan-Gomez C Bombardieri CR . Restricted diet delays accelerated ageing and genomic stress in DNA-repair-deficient mice. Nature. (2016) 537:427–31. 10.1038/nature19329 27556946 PMC5161687

[27] Wardill HR da Silva Ferreira AR Lichtenberg Cloo S Havinga R Harmsen HJM Vermeij WP . Pre-therapy fasting slows epithelial turnover and modulates the microbiota but fails to mitigate methotrexate-induced gastrointestinal mucositis. Gut Microbes. (2020) 12:1–9. 10.1080/19490976.2020.1809332 32844722 PMC7524354

[28] de Cabo R Mattson MP. Effects of intermittent fasting on health, aging, and disease. N Engl J Med. (2019) 381:2541–51. 10.1056/NEJMra1905136 31881139

[29] Redman LM Heilbronn LK Martin CK Alfonso A Smith SR Ravussin E . Effect of calorie restriction with or without exercise on body composition and fat distribution. J Clin Endocrinol Metab. (2007) 92:865–72. 10.1210/jc.2006-2184 17200169 PMC2692618

[30] Rochon J Bales CW Ravussin E Redman LM Holloszy JO Racette SB . Design and conduct of the CALERIE study: comprehensive assessment of the long-term effects of reducing intake of energy. J Gerontol A Biol Sci Med Sci. (2011) 66:97–108. 10.1093/gerona/glq168 20923909 PMC3032519

[31] Jongbloed F Bruin RWFd Pennings JLA Payan-Gomez C Engel Svd Oostrom CTMv . Preoperative fasting protects against renal ischemia-reperfusion injury in aged and overweight mice. PLoS ONE. (2014) 9:e100853. 10.1371/journal.pone.0100853 24959849 PMC4069161

[32] Chen G Bridenbaugh EA Akintola AD Catania JM Vaidya VS Bonventre JV . Increased susceptibility of aging kidney to ischemic injury: identification of candidate genes changed during aging, but corrected by caloric restriction. Am J Physiol Renal Physiol. (2007) 293:F1272–81. 10.1152/ajprenal.00138.2007 17670906 PMC2758575

[33] Jongbloed F Saat TC Verweij M Payan-Gomez C Hoeijmakers JHJ Engel Svd . A signature of renal stress resistance induced by short-Term dietary restriction, fasting, and protein restriction. Sci Rep. (2017) 7:40901. 10.1038/srep40901 28102354 PMC5244361

[34] Shushimita S Pol Pvd Bruin RWFd IJzermans JNM Kooten Cv Dor FJMF. Mannan-binding lectin is involved in the protection against renal ischemia/ reperfusion injury by dietary restriction. PLoS ONE. (2015) 10:e0137795. 10.1371/journal.pone.0137795 26367533 PMC4569339

[35] van Ginhoven TM de Bruin RW Timmermans M Mitchell JR Hoeijmakers JH Ijzermans JN. Pre-operative dietary restriction is feasible in live-kidney donors. Clin Transplant. (2011) 25:486–94. 10.1111/j.1399-0012.2010.01313.x 20718826

[36] Jongbloed F Bruin RWFd Klaassen RA Beekhof P Steeg Hv Dor FJMF . Short-term preoperative calorie and protein restriction is feasible in healthy kidney donors and morbidly obese patients scheduled for surgery. Nutrients. (2016) 8:306. 10.3390/nu8050306 27213441 PMC4882718

[37] Jongbloed F de Bruin RWF Steeg HV Beekhof P Wackers P Hesselink DA . Protein and calorie restriction may improve outcomes in living kidney donors and kidney transplant recipients. Aging (Albany NY). (2020) 12: 12441–67. 10.18632/aging.103619 32652516 PMC7377854

[38] de Groot S Lugtenberg RT Cohen D Welters MJP Ehsan I Vreeswijk MPG . Fasting mimicking diet as an adjunct to neoadjuvant chemotherapy for breast cancer in the multicentre randomized phase 2 DIRECT trial. Nat Commun. (2020) 11:3083. 10.1038/s41467-020-16138-3 32576828 PMC7311547

[39] Mitchell JR Verweij M Brand K Ven HWMvd Goemaere NNT Engel Svd . Short-term dietary restriction and fasting precondition against ischemia reperfusion injury in mice. Aging Cell. (2010) 9:40–53. 10.1111/j.1474-9726.2009.00532.x 19878145 PMC3412229

[40] Schumacher B van der Pluijm I Moorhouse MJ Kosteas T Robinson AR Suh Y . Delayed and accelerated aging share common longevity assurance mechanisms. PLoS Genet. (2008) 4:e1000161. 10.1371/journal.pgen.1000161 18704162 PMC2493043

[41] Finkel T. The metabolic regulation of aging. Nat Med. (2015) 21:1416–23. 10.1038/nm.3998 26646498

[42] Caputo M Pigni S Agosti E Daffara T Ferrero A Filigheddu N . Regulation of GH and GH Signaling by Nutrients. Cells. (2021) 10:1376. 10.3390/cells10061376 34199514 PMC8227158

[43] Blake NG Eckland DJ Foster OJ Lightman SL. Inhibition of hypothalamic thyrotropin-releasing hormone messenger ribonucleic acid during food deprivation. Endocrinology. (1991) 129:2714–8. 10.1210/endo-129-5-2714 1935800

[44] Kalogeris T Baines CP Krenz M Korthuis RJ. Cell biology of ischemia/reperfusion injury. Int Rev Cell Mol Biol. (2012) 298:229–317. 10.1016/B978-0-12-394309-5.00006-7 22878108 PMC3904795

[45] Saat TC Akker EKvd IJzermans JNM Dor FJMF Bruin RWFd. Improving the outcome of kidney transplantation by ameliorating renal ischemia reperfusion injury: Lost in translation? J Transl Med. (2016) 14:20. 10.1186/s12967-016-0767-2 26791565 PMC4721068

[46] van Ginhoven TM Mitchell JR Verweij M Hoeijmakers JH Ijzermans JN de Bruin RW. The use of preoperative nutritional interventions to protect against hepatic ischemia-reperfusion injury. Liver Transpl. (2009) 15:1183–91. 10.1002/lt.21871 19790167

[47] Knijnenburg SL Jaspers MW van der Pal HJ Schouten-van Meeteren AY Bouts AH Lieverst JA . Renal dysfunction and elevated blood pressure in long-term childhood cancer survivors. Clin J Am Soc Nephrol. (2012) 7:1416–27. 10.2215/CJN.09620911 22822016 PMC3430951

[48] Dekkers IA Blijdorp K Cransberg K Pluijm SM Pieters R Neggers SJ . Long-term nephrotoxicity in adult survivors of childhood cancer. Clin J Am Soc Nephrol. (2013) 8:922–9. 10.2215/CJN.09980912 23411430 PMC3675853

[49] Hui-Stickle S Brewer ED Goldstein SL. Pediatric ARF epidemiology at a tertiary care center from 1999 to 2001. Am J Kidney Dis. (2005) 45:96–101. 10.1053/j.ajkd.2004.09.028 15696448

[50] Interiano RB Delos Santos N Huang S Srivastava DK Robison LL Hudson MM . Renal function in survivors of nonsyndromic Wilms tumor treated with unilateral radical nephrectomy. Cancer. (2015) 121:2449–56. 10.1002/cncr.29373 25832759 PMC5161342

[51] Green DM Wang M Krasin M Srivastava D Onder S Jay DW . Kidney function after treatment for childhood cancer: a report from the St. Jude Lifetime Cohort Study. J Am Soc Nephrol. (2021) 32:983–93. 10.1681/ASN.2020060849 33653686 PMC8017532

[52] Romao RL Lorenzo AJ. Renal function in patients with Wilms tumor. Urol Oncol. (2016) 34:33–41. 10.1016/j.urolonc.2015.07.002 26278364

[53] Stefanowicz J Kosiak M Kosiak W Lipska-Zietkiewicz BS. Chronic kidney disease in Wilms tumour survivors – what do we know today? In: van den Heuvel-Eibrink MM, editor. Wilms Tumor Brisbane (AU): Codon Publications (2016). 27512760

[54] Neu MA Russo A Wingerter A Alt F Theruvath J El Malki K . Prospective analysis of long-term renal function in survivors of childhood Wilms tumor. Pediatr Nephrol. (2017) 32:1915–25. 10.1007/s00467-017-3673-9 28451896

[55] Muzaale AD Massie AB Wang MC Montgomery RA McBride MA Wainright JL . Risk of end-stage renal disease following live kidney donation. JAMA. (2014) 311:579–86. 10.1001/jama.2013.285141 24519297 PMC4411956

[56] O'Keeffe LM Ramond A Oliver-Williams C Willeit P Paige E Trotter P . Mid- and Long-term health risks in living kidney donors: a systematic review and meta-analysis. Ann Intern Med. (2018) 168:276–84. 10.7326/M17-1235 29379948

[57] Garg AX Suri RS Barrowman N Rehman F Matsell D Rosas-Arellano MP . Long-term renal prognosis of diarrhea-associated hemolytic uremic syndrome: a systematic review, meta-analysis, and meta-regression. JAMA. (2003) 290:1360–70. 10.1001/jama.290.10.1360 12966129

[58] Askenazi DJ Feig DI Graham NM Hui-Stickle S Goldstein SL. 3-5 year longitudinal follow-up of pediatric patients after acute renal failure. Kidney Int. (2006) 69:184–9. 10.1038/sj.ki.5000032 16374442

[59] Sutherland SM Ji J Sheikhi FH Widen E Tian L Alexander SR . AKI in hospitalized children: epidemiology and clinical associations in a national cohort. Clin J Am Soc Nephrol. (2013) 8:1661–9. 10.2215/CJN.00270113 23833312 PMC3789331

[60] Sutherland SM Byrnes JJ Kothari M Longhurst CA Dutta S Garcia P . AKI in hospitalized children: comparing the pRIFLE, AKIN, and KDIGO definitions. Clin J Am Soc Nephrol. (2015) 10:554–61. 10.2215/CJN.01900214 25649155 PMC4386245

[61] van Veen MR van Hasselt PM de Sain-van der Velden MG Verhoeven N Hofstede FC de Koning TJ . Metabolic profiles in children during fasting. Pediatrics. (2011) 127:e1021–7. 10.1542/peds.2010-1706 21422093

[62] Bastiaansen D Koot HM Bongers IL Varni JW Verhulst FC. Measuring quality of life in children referred for psychiatric problems: psychometric properties of the PedsQL 4. 0 generic core scales. Qual Life Res. (2004) 13:489–95. 10.1023/B:QURE.0000018483.01526.ab 15085921

[63] Varni JW. The PedsQL Measurement Model for the Pediatric Quality of Life Inventory. Available online from: https://www.pedsql.org/ (accessed December 22, 2021). 10.1097/00005650-199902000-00003 10024117

[64] Ida S Kaneko R Murata K. SARC-F for screening of sarcopenia among older adults: a meta-analysis of screening test accuracy. J Am Med Dir Assoc. (2018) 19:685–9. 10.1016/j.jamda.2018.04.001 29778639

[65] Connolly B MacBean V Crowley C Lunt A Moxham J Rafferty GF . Ultrasound for the assessment of peripheral skeletal muscle architecture in critical illness: a systematic review. Crit Care Med. (2015) 43:897–905. 10.1097/CCM.0000000000000821 25559437

[66] McCarthy HD Samani-Radia D Jebb SA Prentice AM. Skeletal muscle mass reference curves for children and adolescents. Pediatr Obes. (2014) 9:249–59. 10.1111/j.2047-6310.2013.00168.x 23776133

[67] Harvey AR. The Gross Motor Function Measure (GMFM). J Physiother. (2017) 63:187. 10.1016/j.jphys.2017.05.007 28633883

[68] Khwaja A. KDIGO clinical practice guidelines for acute kidney injury. Nephron Clin Pract. (2012) 120:c179–84. 10.1159/000339789 22890468

[69] Zhang J Han J Liu J Liang B Wang X Wang C. Clinical significance of novel biomarker NGAL in early diagnosis of acute renal injury. Exp Ther Med. (2017) 14:5017–21. 10.3892/etm.2017.5150 29201207 PMC5704279

[70] Ichimura T Hung CC Yang SA Stevens JL Bonventre JV. Kidney injury molecule-1: a tissue and urinary biomarker for nephrotoxicant-induced renal injury. Am J Physiol Renal Physiol. (2004) 286:F552–63. 10.1152/ajprenal.00285.2002 14600030

[71] Migueles JH Cadenas-Sanchez C Ekelund U Delisle Nyström C Mora-Gonzalez J Löf M . Accelerometer data collection and processing criteria to assess physical activity and other outcomes: a systematic review and practical considerations. Sports Med. (2017) 47:1821–45. 10.1007/s40279-017-0716-0 28303543 PMC6231536

[72] Fleiss JL Levin B Paik MC. Statistical methods for rates and proportions. 3rd ed. Hoboken: John Wiley & Sons (2003). p. 26

[73] Dindo D Demartines N Clavien PA. Classification of surgical complications: a new proposal with evaluation in a cohort of 6336 patients and results of a survey. Ann Surg. (2004) 240:205–13. 10.1097/01.sla.0000133083.54934.ae 15273542 PMC1360123

[74] Dennhardt N Beck C Huber D Nickel K Sander B Witt LH . Impact of preoperative fasting times on blood glucose concentration, ketone bodies and acid-base balance in children younger than 36 months: a prospective observational study. Eur J Anaesthesiol. (2015) 32:857–61. 10.1097/EJA.0000000000000330 26351828

