# Supplementary figures and images for: Fasting Intervention for Children With Unilateral Renal Tumors to Reduce Toxicity
Source: Front Pediatr. 2022 Jan 27;10:828615. doi: 10.3389/fped.2022.828615 (PMC8829466; doi:10.3389/fped.2022.828615)

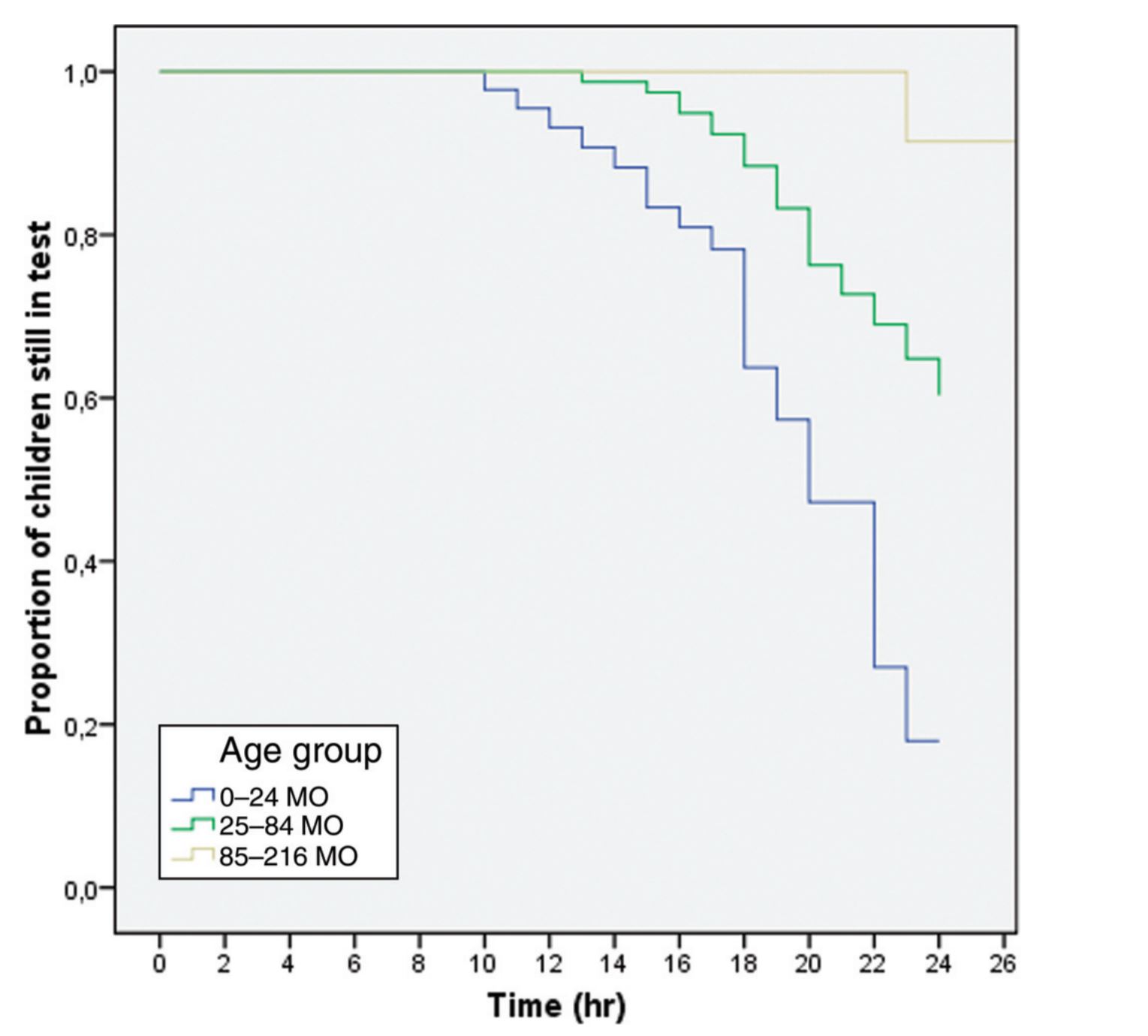

Supplement: Supplementary Figure 1 — Schematic overview of the FIURTT study. [file Image_1.JPEG]

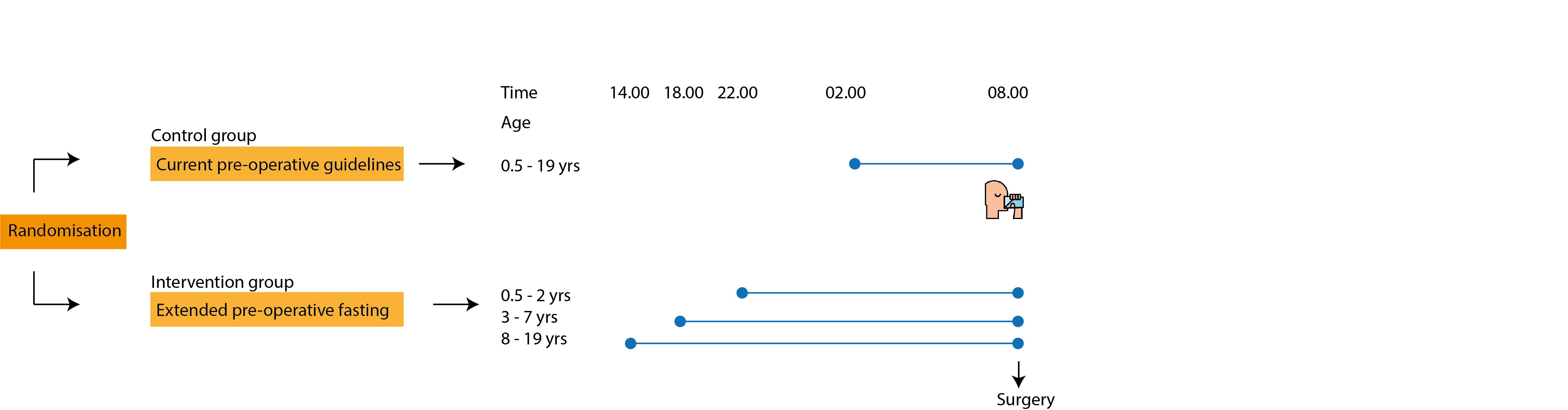

Supplement: Supplementary Figure 2 — Survival graphic from the fasting test from van Veen et al. (61). [file Image_2.JPEG]
