# Supplementary material for: Fasting Intervention for Children With Unilateral Renal Tumors to Reduce Toxicity
Source: Front Pediatr. 2022 Jan 27;10:828615. doi: 10.3389/fped.2022.828615 (PMC8829466; doi:10.3389/fped.2022.828615)
Supplement: Supplementary file 3 [file Data_Sheet_1.docx]

# Supplementary Materials

A Figure 1: Schematic overview of the FIURTT study

B Figure 2: Survival graphic from the fasting test from Van Veen et al (1)

C Informed Consent Forms:

- Patient information letter and Informed Consent form – parents/caretakers
- Patient information letter and Informed Consent form patients 12 -16 years
- Patient information letter and Informed Consent form patients older than 16 years.

## Appendices

A Plans for collection, laboratory evaluation, and storage of biological specimens for genetic or molecular analysis in the current trial and for use in future studies

B Study Workflow

# Appendix A

Overview of biological specimens:

- Healthy renal tissue
  - Biopsy 1, 2 and 3: 5x5x5 mm
- Renal tumour tissue
  - Biopsy 1, 2 and 3: 5x5x5 mm
- Ureter Tissue
  - Biopsy 1, 2 and 3: +/- 1 cm length, depends on primary sample
- Blood sample, 6 mL

## Acquiring tissue samples

To obtain markers of the protective stress response, several tissue biopsies are performed after surgical removal of the renal tumour. These biopsies, acquired to investigate the tissue and tumour, are part of the routine procedure. No additional tissues are biopsied, and the healthy (contralateral) kidney is left untouched. The following tissue samples are acquired:

- According to SIOP-protocol: (intact) renal tumour. Removed *in toto* during surgery, pathological histological assessment and international review will be performed according to SIOP RTSG-UMBRELLA -protocol
- Healthy renal tissue of the ipsilateral kidney (which will be removed *in toto*)
- Leftover ipsilateral ureter tissue

The tumour is left undisturbed until the pathologist will conduct the standard procedure according to SIOP-protocol. Biopsy will be performed by the pathologist, after surgery. This biopsy will be flash-frozen in liquid nitrogen as quick as possible. It is important to note that this biopsy will only be acquired from tissue which would otherwise be removed from the subject as well. Therefore, these biopsies do not cause any additional risk to the diagnostic procedure. Analysis of the tumour, including determination of the amount of necrosis, is done according to standard protocol as well (2-4).

The before-mentioned kidney tissue, tumour and ureter biopsies are assessed for molecular and cytoprotective markers associated with the fasting diet and determine critical parameters of induction of the ‘survival response’. In previous studies using mice, we have determined a panel of cytoprotective genes activated during fasting regimens (5, 6). These and other potentially interesting genes will be determined via RNA expression analysis.

A part of the biopsies will be snap-frozen and fixed in formaldehyde for immuno-histochemistry for determining e.g., DNA damage parameters or oxidative stress signalling. These include overall thiol (SS/SH) oxidation state analysed by redox western blots, DNA damage levels using yH2AX foci analysis, and inflammatory state using gene expression analysis.

Remaining histological material investigated for this study will be stored at Princess Maxima Center for Pediatric Oncology Biobank during study runtime. After conclusion of our study, histologic material will be stored at Princess Maxima Center for Pediatric Oncology Biobank with a minimal retention period of 15 years, if subjects signed the separate consent, in accordance with current laws and regulations. Material will then be available for additional research on this topic. Subjects are not obligated to sign this separate informed consent for inclusion into this study, if they do not sign the separate consent, their remaining histological material will be discarded appropriately at study conclusion.

## Analysis of blood & tissue samples

### DR/Fasting + damage markers

GH, GHR, IGF-I, DIO1, DIO3, leptin, ghrelin, IGFBPs, ACOT1, RRM1, EGR1, IGF-1 receptor, FGF-21, GDF-15, TRIM24, SIRT1, ENT1, NRF2, HO-1, PGC-1a, ELOVL3, p16, p21, p53, IL-6, TNFa

Total RNA will be isolated from tissue specimens for analysis of DR/Fasting and DNA damage response markers using qPCR or RNA-seq. Expression data of markers will be correlated to blood values from diagnostics laboratory. Where possible, results will be further assessed at a protein level using western blotting.

### Immunohistochemistry/Immunofluorescence

- Senescence: HMGB1, lamin B1, p21
- DNA damage: yH2AX, 53BP1, RAD51
- Apoptosis: TUNEL, cleaved caspase-3

### Redox western blot

Assay for monitoring thiol redox states, which are known to change upon caloric restriction/fasting. With use of chemical modifiers that can specifically bind covalently with free thiol groups, thereby inactivating them. Reducing agents cannot remove these modifications. After subsequent reduction of oxidized thiol groups, which were engaged in disulfide bonds, these could be labelled with a second compound. These can then be analysed using mass spectrometry, western blotting, or histology.

### Organotypic slices

Method described for several tissue types, but generally as follows: Tissue samples should be obtained as quick as possible but no later than 4 hours after surgical resection and kept at 4°C during transport to the laboratory. Keep tissue sample in culture medium while transported. If required, remove excess fat from tissue sample and make slices using the vibratome (*thickness of slices should be determined beforehand, 200-300 µm*). Slices should be transferred to wells plate/culture dish containing culture medium. Tissues should be incubated at 37C, 5% CO2, on a shaking platform (*60 rpm*). Additional assays (e.g., EU/EdU incubation) can be performed while slices are in culture. Tissue slices can be fixed in paraformaldehyde or formalin and further sliced using the microtome. Material can then be used for immunohistochemistry or immunofluorescence.

# References

1. van Veen MR, van Hasselt PM, de Sain-van der Velden MG, Verhoeven N, Hofstede FC, de Koning TJ, et al. Metabolic profiles in children during fasting. Pediatrics. 2011;127(4):e1021-7.

2. Huff V. Wilms' tumours: about tumour suppressor genes, an oncogene and a chameleon gene. Nat Rev Cancer. 2011;11(2):111-21.

3. Brok J, Treger TD, Gooskens SL, van den Heuvel-Eibrink MM, Pritchard-Jones K. Biology and treatment of renal tumours in childhood. Eur J Cancer. 2016;68:179-95.

4. Beckwith JB, Kiviat NB, Bonadio JF. Nephrogenic rests, nephroblastomatosis, and the pathogenesis of Wilms' tumor. Pediatr Pathol. 1990;10(1-2):1-36.

5. Schumacher B, van der Pluijm I, Moorhouse MJ, Kosteas T, Robinson AR, Suh Y, et al. Delayed and accelerated aging share common longevity assurance mechanisms. PLoS genetics. 2008;4(8):e1000161.

6. Jongbloed F, de Bruin RWF, Steeg HV, Beekhof P, Wackers P, Hesselink DA, et al. Protein and calorie restriction may improve outcomes in living kidney donors and kidney transplant recipients. Aging (Albany NY). 2020;12.

# Appendix B: Study Workflow: Overview of study runtime and measurements included in the FIURTT study

| **Study Day (0 = Surgery, in days)** | **T = -28** | **T = -21** | **T = -14** | **T = -7** | **T = -1** | **Surgery**  **T = 0** | **T = +1** | **T = +2** | **T = +3** | **T = +4^1^** | **T = +5^1^** | **T = +8** | **T = +28** |
| --- | --- | --- | --- | --- | --- | --- | --- | --- | --- | --- | --- | --- | --- |
| Location:  OC: Outpatient Clinic, HA: Hospital Admittance | **OC** | **OC** | **OC** | **OC** | **HA** | **HA** | **HA** | **HA** | **HA** | **HA** | **HA** | **OC** | **OC** |
| Chemotherapy | **Cycle 1** | **Cycle 2** | **Cycle 3** | **Cycle 4** | **-** | **-** | **-** | **-** | **-** | **-** | **-** | **-** | **-** |
| **Informed Consent** |  |  | **X** |  |  |  |  |  |  |  |  |  |  |
| **Inclusion & Randomisation** |  |  |  | **X** |  |  |  |  |  |  |  |  |  |
| **Measurements:** |  |  |  |  |  |  |  |  |  |  |  |  |  |
| - Quality of Life | **-** | **-** | **-** | **-** | **x** | **-** | **-** | **-** | **-** | ***-*** | **x** | **x** | **-** |
| - Pain^5^ | **-** | **-** | **-** | **-** | **x** | **x** | **x** | **x** | **x** | **x** | **x** | **x** | **-** |
| - Accelerometer^5^ | **-** | **-** | **-** | **x** | **x** | **-** | **-** | **-** | **-** | **x** | **-** | **x** | **-** |
| - Physical assessment^5^ | **-** | **-** | **-** | **x** | **-** | **-** | **-** | **-** | **-** | **-** | **-** | **-** | **x** |
| - Karnofsky / Lansky score^5^ | **-** | **-** | **-** | **-** | **x** | **x** | **x** | **x** | **x** | **x** | **x** | **x** | **-** |
| - Weight^5^ | **x ^2^** | **x ^2^** | **x ^2^** | **x** | **x** | **x** | **x** | **x** | **x** | **x** | **x** | **x** | **x** |
| - Height^5^ | **x ^2^** | **x ^2^** | **x ^2^** | **x** | **x** | **x** | **x** | **x** | **x** | **x** | **x** | **x** | **x** |
| **Laboratory testing** |  |  |  |  |  |  |  |  |  |  |  |  |  |
| - Blood ^3^ | **-** | **-** | **-** | **-** | **x** | **x** | **x** | **x** | **x** | **x** | **x** | **x** | **x** |
| - Urine ^4^ | **-** | **-** | **-** | **-** | **-** | **x** | **x** | **x** | **x** | **-** | ***-*** | **-** | **x** |
| **Biopsy** |  |  |  |  |  |  |  |  |  |  |  |  |  |
| - Ureter | **-** | **-** | **-** | **-** | **-** | **x** | **-** | **-** | **-** | **-** | **-** | **-** | **-** |
| - Healthy Renal Tissue | **-** | **-** | **-** | **-** | **-** | **x** | **-** | **-** | **-** | **-** | **-** | **-** | **-** |
| - Tumour specimen | **-** | **-** | **-** | **-** | **-** | **x** | **-** | **-** | **-** | **-** | **-** | **-** | **-** |

Table 1: Study overview / timing of measurements

***1:** Duration of hospital stay depends on postoperative recovery.

***2:** Weight and height will be collected from electronic patient file from earlier visits.

***3:** Laboratory testing will be done at marked moments. These moments do not result in additional invasive procedures for withdrawing of blood: subjects already have an intravenous catheter which is part of standard of care. Measured lab values will be values which would also be measured during standard care, except at admittance before starting the diet and on the day of surgery: two additional tubes will be drawn for molecular analysis and determination of metabolic parameters.

***4:** Urine sampling will be done via catheter during admission, which is placed according to current practice. Urine sampling on T = 28 days will be done without catheter.

***5:** Both are used during Standard Care, but the timing will be slightly changed to coincide with the study.
