# Supplementary material for: Fasting Intervention for Children With Unilateral Renal Tumors to Reduce Toxicity
Source: Front Pediatr. 2022 Jan 27;10:828615. doi: 10.3389/fped.2022.828615 (PMC8829466; doi:10.3389/fped.2022.828615)
Supplement: Supplementary file 4 [file Data_Sheet_2.pdf]

Informatiebrief met toestemmingsformulier voor jongeren (≥16 jaar) voor deelname aan medisch wetenschappelijk onderzoek: de FIURTT Studie

Vasten voor de operatie en het herstel na een operatie voor een niertumor
Officiële titel: Kortdurend vasten voor kinderen met unilaterale niertumoren ter vermindering van toxiciteit en bevorderen postoperatief herstel

Inleiding

Beste,

Wij vragen je om mee te doen aan een medisch-wetenschappelijk onderzoek. Meedoen is vrijwillig. Om mee te doen is wel jouw schriftelijke toestemming nodig.

Voordat je beslist of je mee wilt doen aan dit onderzoek, krijg je uitleg over wat het onderzoek inhoudt. Lees deze informatie rustig door en vraag de dokter of onderzoeker uitleg als je vragen hebt. Je kunt ook de onafhankelijk deskundige, die aan het eind van deze brief genoemd wordt, om aanvullende informatie vragen. Je kan er ook over praten met je ouders, broers/zussen, vrienden of familie.

Verdere informatie over meedoen aan zo'n onderzoek staat op de online pagina 'Medisch-wetenschappelijk onderzoek'. Deze pagina kan je vinden via https://www.rijksoverheid.nl/onderwerpen/medisch-wetenschappelijk-onderzoek. Er is ook een folder van de VKN (Vereniging Kinderkanker Nederland) over klinisch onderzoek. Deze folder zit in de dagboekagenda die je aan het begin van de behandeling krijgt.

Hebben jij of jouw ouders na het lezen van de informatie nog vragen? Dan kan je terecht bij de behandelend dokter of de researchverpleegkundige. In Bijlage A vind je de contactgegevens.

Jij en jouw ouders beslissen samen of je meedoet of niet.

1. Algemene informatie

Je ontvangt deze brief omdat je onder behandeling bent op de afdeling kinderoncologie voor een niertumor. Tijdens deze behandeling krijg je eerst 4 weken lang 1 dag in de week chemotherapie, aansluitend zal een operatie plaatsvinden in de 5^{e} of 6^{e} week. Wij willen onderzoeken hoe we jou zo goed mogelijk kunnen laten herstellen na de operatie.

Dit onderzoek is opgezet en wordt verricht door het Prinses Máxima Centrum voor Kinderoncologie. Het Prinses Máxima Centrum voor Kinderoncologie vergoedt de kosten van dit onderzoek. Voor dit onderzoek zijn ongeveer vijftig kinderen met een niertumor in de leeftijd van 6 maanden tot 19 jaar nodig.

De medisch-ethische toetsingscommissie Utrecht heeft dit onderzoek goedgekeurd. Algemene informatie over de toetsing van onderzoek vind je op de onlinepagina 'Medisch-wetenschappelijk onderzoek'.

2. Doel van het onderzoek

Het doel van dit onderzoek is om te onderzoeken of vasten voor de operatie in verband met een niertumor ervoor zorgt dat je na de operatie sneller en beter herstelt. Normaal moet je ook nuchter zijn voor een operatie, maar vasten houdt in dat je langer (een aantal uur) voor je operatie niet mag eten. Wij willen onderzoeken of, als je een korte periode niet eet, je lichaam zich in de "beschermstand" zet. Deze "beschermstand" zorgt er mogelijk voor dat je beter tegen de lichamelijke stress (oftewel schade) van de operatie kan. Het vasten zou er zo voor kunnen zorgen dat je sneller herstelt na de operatie en dat er minder schade is in de nier die je blijft houden. Dit willen we onderzoeken bij deze ziekte en de bijbehorende operatie. Afhankelijk van de resultaten van het onderzoek willen we het vervolgens toepassen in de dagelijkse zorg.

3. Achtergrond van het onderzoek

Verschillende onderzoeken hebben laten zien dat dieren langer leven als er minder calorieën in de voeding zitten dan normaal. Dit komt gedeeltelijk door een betere weerstand tegen oxidatieve stress, weerstand die het lichaam zelf opbouwt als er minder calorieën in de voeding zitten. Oxidatieve stress is een soort schade die ontstaat in situaties die schadelijk zijn voor het lichaam zoals warmte, UV-licht en roken. Oxidatieve stress ontstaat ook tijdens een operatie en kan nadelige invloed hebben op het herstel na de operatie.

Eerder onderzoek met nierdonoren die een nier bij leven hebben afgestaan en patiënten die een maagverkleining hebben ondergaan lieten zien dat een dieet danwel vasten voor de operatie goed werd verdragen zonder een hoger risico op complicaties na de operatie. Ook werd er een mogelijk effect op beter herstel bij de donor en de ontvanger van de nier gezien. Nu willen we onderzoeken of dit gunstige effect ook aanwezig is bij kinderen die worden geopereerd aan een niertumor.

4. Wat meedoen inhoudt

Als je meedoet met dit onderzoek, zal het onderzoek aanvullend aan de standaardbehandeling voor de niertumor plaatsvinden. Het eerste deel van de standaardbehandeling voor niertumoren neemt ongeveer 6 weken in beslag. Dit bestaat uit 1 maand chemotherapie (4 keer een cyclus van 1 week), een operatie en het herstel na de operatie.

Het vasten vindt plaats vanaf 1 dag voor de operatie en dit duurt tot en met de operatie. Na de operatie mag je weer eten afhankelijk van hoe de operatie gegaan is.

Geschiktheidsonderzoek
Op basis van jouw medische gegevens zal gekeken worden, in samenspraak met je behandelend arts, of je mee kan doen aan het onderzoek. Hiervoor zijn geen extra onderzoeken nodig.

Het onderzoek
Wanneer jij toestemming geeft en meedoet aan dit onderzoek, kom je via willekeurige loting in één van twee onderzoeksgroepen. Dit betekent dat je 50% kans hebt om in de vastengroep geloot te worden.

<u>Groep 1:</u>
De standaardbehandeling: je wordt vanaf 6 uur (vanaf 02:00) voor de operatie nuchter gehouden. Nuchter zijn betekent geen vast eten. Je mag tot vlak voor de operatie nog wel heldere suikerhoudende dranken drinken.

<u>Groep 2:</u>
De behandeling die we onderzoeken: je wordt 18 uur (vanaf 14:00) voor de operatie nuchter gehouden. Je mag niet eten. Tot vlak voor de operatie mag er wel suikervrije limonade, water of thee gedronken worden, als er maar geen calorieën in zitten (dus geen suiker in de thee).

Het vasten start pas als je bent opgenomen in het ziekenhuis, zie ook bijlage B.

Wat is er meer of anders dan de gebruikelijke zorg of behandeling?
Normaal kom je ongeveer 1 keer per week bij de behandelend dokter en duurt een controle een half uur. De bezoeken die bij dit onderzoek horen, vinden plaats op het moment van de normale controles en daardoor kunnen de controles 15-20 minuten langer duren. Als je ingeloot wordt voor de behandeling die we onderzoeken, dan mag je voor de operatie voor een aantal uren langer durende periode niet eten. Zie de volgende alinea voor wat er precies extra gebeurt in verband met het onderzoek.

Bezoeken en metingen
Voor het onderzoek zijn geen extra afspraken nodig in het ziekenhuis. Jij en jouw ouders komen op de reguliere afspraakmomenten langs. Wel is het mogelijk dat de afspraak iets langer duurt als je meedoet aan dit onderzoek, dit is maximaal 15 tot 20 minuten langer dan als je niet meedoet.

In het eerste deel van je behandeling kom je wekelijks naar het ziekenhuis voor de medicijnbehandeling. In de derde week van de medicijnbehandeling gaat dit onderzoek lopen en gaan we <u>extra</u> gegevens verzamelen.

Dit zijn:

- Voor de operatie, tijdens de ziekenhuisopname en 2-3 dagen na de opname willen we door middel van een klein apparaatje meten hoeveel je beweegt/hoe actief je bent. Dit kleine apparaatje zit op een soort riem die om je heup zit. Dit apparaatje wordt al in het ziekenhuis gebruikt. In verband met dit onderzoek krijg je dit apparaatje voor de operatie alvast mee naar huis om te oefenen met het dragen en om te kijken hoe actief je bent voor de operatie. Als je het apparaatje draagt heb je er weinig last van, je kan alles doen wat je normaal ook zou doen. Onze kinderfysiotherapeut zal je helpen met hoe het apparaatje werkt en hoe je het moet gebruiken.
- Voor en na de operatie krijg je een aanvullende afspraak bij de kinderfysiotherapeut. Tijdens deze afspraak van ongeveer 20 minuten zullen verschillende metingen verricht worden t.a.v. je lichamelijke conditie.
- Voor de operatie wordt het bloedsuikergehalte gecontroleerd. Dit gebeurt door middel van een vingerprik. Dit is een kleine prik waar je weinig last van hebt.
- Bij de nieroperatie krijg je altijd een infuus voor de operatie, je krijgt hier medicijnen door, maar er wordt ook meermaals bloed via afgenomen. We willen hier twee keer één extra buisje bloed uit afnemen tijdens een normaal afnamemoment. We nemen bloed af uit je infuuslijn om te meten hoe je lichaam reageert op het vasten. Je krijgt dus geen extra prik.
- Rondom de operatie krijg je altijd een slangetje in je urinebuis (een katheter). Daar nemen we een aantal dagen urine uit af voor onderzoek. Ook vragen we aan jou om je urine mee te nemen als je na 4 weken op de polikliniek terugkomt.
- Jij en jouw ouders krijgen 3x een vragenlijst over hoe het met je gaat. De vragenlijst kost je per keer ongeveer 10-15 minuten, de vragenlijst wordt digitaal ingevuld via het KLIK-Portaal van het Prinses Máxima Centrum. Dit doen we om na te gaan hoe belastend het vasten voor jou en je ouders is.
- Er wordt door een patholoog (een arts die gespecialiseerd is in het onderzoek van lichaamsweefsel en cellen) een stukje afgenomen van je zieke nier tijdens het weefselonderzoek na de operatie. Dit weefsel wordt gebruikt om te onderzoeken hoe de cellen hebben gereageerd op het vasten. Hier ondervind je geen last van.

Er is ook informatie die we in verband met het onderzoek gebruiken maar die we al verzamelen als onderdeel van de standaardbehandeling:

- We gaan je gewicht en lengte dagelijks meten als je in het ziekenhuis bent.
- Alle andere bloedafnames gaan door zoals dat normaal ook gaat.
- We houden de urineproductie bij zoals dat normaal ook gaat.

5. Wat wordt er van jou verwacht

Om het onderzoek goed te laten verlopen en voor jouw veiligheid, is het belangrijk dat jij en jouw ouders zich aan de volgende afspraken houden:

De afspraken zijn dat jij en jouw ouders:

- Het vastendieet volgen volgens voorschrift/uitleg tijdens de opname.
- De vragenlijsten invullen.
- De activiteitenmeter gebruiken zoals dat is uitgelegd.
- Niet ook nog aan een ander medisch-wetenschappelijk onderzoek meedoen zonder medeweten van de onderzoeker.
- De afspraken voor bezoeken zo goed mogelijk nakomen.

Het is belangrijk dat jij of jouw ouders contact opnemen met de onderzoeker of researchverpleegkundige:

- Voordat je andere geneesmiddelen gaat gebruiken. Ook als dat homeopathische geneesmiddelen, natuurgeneesmiddelen, vitaminen en/of geneesmiddelen van de drogist zijn.
- Als je in een ziekenhuis wordt opgenomen of behandeld.
- Als je plotseling gezondheidsklachten krijgt.
- Als jij niet meer mee wilt doen aan het onderzoek.
- Als je contactgegevens wijzigen.

Je krijgt een kaartje met telefoonnummers en informatie over het onderzoek. Wij vragen u om dit kaartje mee te nemen bij elk ziekenhuisbezoek.

6. Mogelijke complicaties en eventuele nadelige effecten

Het (langer) vasten in het kader van het onderzoek kan neveneffecten geven. Je kan tijdens het vasten mogelijk een hongergevoel ervaren of licht draaierig worden. Is dit het geval, dan kan je dit aangeven bij de verpleegkundige en/of afdelingsarts. Daarnaast is het vasten een verandering in het dagelijks leven en kan het zodoende gepaard gaan met stress en/of onwennigheid. In zeldzame gevallen kan je een te laag bloedsuikergehalte krijgen. Wij schatten echter in dat de kans hierop klein is, gezien de gekozen vastentijden. Het bloedsuikergehalte zullen we tijdens het vasten zekerheidshalve controleren. Dit kunnen we goed behandelen met een glucose infuus.

7. Mogelijke voor- en nadelen

Het is belangrijk dat je de mogelijke voor- en nadelen goed afweegt voordat je besluit mee te doen.

Het onderzoek kijkt naar de effecten van een vastendieet op het herstel na een operatie. Eerdere onderzoeken naar dit onderwerp lieten geen nadelige effecten maar juist enkele voordelige effecten zien. Deze vastentherapie voor een operatie voor een niertumor kan een gunstig effect geven, maar zeker is dat niet. We verwachten dat dit onderzoeken in de toekomst bijdraagt aan beter herstel na een operatie. Jij draagt bij aan meer kennis over de behandeling van niertumoren voor andere kinderen/jongeren.

Als je deelneemt aan het onderzoek kan je mogelijk neveneffecten verwachten van het vasten. Hierover staat meer onder punt 6. Voor het onderzoek wordt niet een extra bloedafnamemoment verricht, maar er wordt wel tweemaal één extra buisje bloed afgenomen tijdens een standaard bloedafnamemoment. Je ondervindt hier geen last van. Ook zal je eenmaal wat extra urine moeten inleveren. Indien je ingeloot wordt voor de behandeling die we onderzoeken, dan mag je voor de operatie gedurende een langere periode niet eten. Het invullen van de vragenlijsten kost je in totaal ongeveer 3 keer 15 minuten in de loop van 2 maanden.

Deelname aan het onderzoek betekent dus ook:
- Dat je tijd kwijt bent aan het invullen van vragenlijsten.
- Dat je thuis een aantal dagen een klein apparaatje op je heup moet dragen.
- Dat je twee extra afspraken krijgt bij de kinderfysiotherapie.
- Dat je afspraken hebt waaraan je je moet houden.
- Dat je eenmalig extra urine en tweemaal één extra buisje bloed moet inleveren voor extra onderzoek.
- Dat wij extra weefselonderzoek verrichten.

Al deze zaken zijn hiervoor onder punt 4, 5 en 6 beschreven.

8. Als je niet mee wilt doen met het onderzoek of als je wilt stoppen met het onderzoek

Jij beslist zelf of je meedoet aan het onderzoek. Deelname is vrijwillig. Als je niet mee wilt doen, word je op de gebruikelijke manier behandeld voor de niertumor. De standaardbehandeling is chemotherapie voor 4 weken met aansluitend een operatie. De nabehandeling hangt af van de operatie en de weefseluitslag, dat is ook geen onderdeel van dit onderzoek.

Als je wel meedoet, kan je je altijd bedenken en stoppen, ook tijdens het onderzoek. De dokter zal dan met jou en jouw ouders bespreken hoe de gewone behandeling en vervolgafspraken gepland worden. Je hoeft niet te zeggen waarom je stopt. Wel moet je dit direct melden aan de behandelend dokter. De gegevens die tot dat moment zijn verzameld, worden gebruikt voor het onderzoek. Als jij of je ouders dit willen, kan verzameld lichaamsmateriaal worden vernietigd.

Als er nieuwe informatie over het onderzoek is die belangrijk voor jou of jouw ouders is, laat de dokter dit aan jullie weten. Er wordt dan gevraagd of je mee blijft doen.

Er is in het Prinses Máxima Centrum een team van gespecialiseerde medewerkers (zoals een dokter, psycholoog, gespecialiseerde verpleegkundige) beschikbaar om je tijdens het onderzoek zo goed mogelijk te begeleiden. Het behandelteam ziet nauwlettend toe op de belasting die deelname aan een onderzoeksprotocol voor jou met zich meebrengt. We werken daarom ook zoveel mogelijk volgens landelijke afspraken zoals die door de Nederlandse Vereniging voor Kindergeneeskunde (NVK) zijn vastgelegd ter bescherming van minderjarige onderzoeksdeelnemers. Voor meer informatie hierover verwijzen we je naar de website www.ccmo.nl onder "wet en regelgeving" gedragscode verzet: minderjarigen.

9. Einde van het onderzoek

Deelname van jou aan het onderzoek stopt:
- Na de poliklinische controleafspraak 4 weken na de operatie.
- Als je zelf kiest om te stoppen.
- Als het einde van het hele onderzoek is bereikt.
- Als de dokter het beter voor je vindt om te stoppen.
- Als de tumor niet te opereren is.
- Als het Prinses Máxima Centrum voor Kinderoncologie, de overheid of de beoordelende medisch-ethische toetsingscommissie, besluit om het onderzoek te stoppen.

Het hele onderzoek is afgelopen als alle deelnemers klaar zijn. De behandelend dokter zal met je praten over de verdere medische zorg na afloop van het onderzoek. Na het verwerken van alle gegevens informeert de onderzoeker je over de belangrijkste uitkomsten van het onderzoek, als je dit wilt.

10. Gebruik en bewaren van de gegevens en lichaamsmaterialen

Voor dit onderzoek worden jouw persoonsgegevens en lichaamsmateriaal verzameld, gebruikt en bewaard. Het gaat om gegevens zoals jouw naam, adres, geboortedatum en om gegevens over jouw gezondheid. Voor dit onderzoek is nierweefsel (tumorweefsel & normaal nierweefsel) nodig. Het verzamelen, gebruiken en bewaren van je gegevens en lichaamsmateriaal gedurende het onderzoek, is nodig om de vragen die in dit onderzoek worden gesteld te kunnen beantwoorden en de resultaten te kunnen publiceren. Het lichaamsmateriaal zal voor analyse opgeslagen worden op de researchafdeling en het laboratorium van het Prinses Máxima Centrum voor Kinderoncologie. Wij vragen voor het gebruik van je gegevens en lichaamsmateriaal jouw toestemming. Als je dat niet wilt, kan je niet deelnemen aan dit onderzoek.

Het lichaamsmateriaal kan na afloop van dit onderzoek ook nog van belang zijn voor ander wetenschappelijk onderzoek op het gebied van het vasten-dieet en operaties. Daarvoor zullen je gegevens en lichaamsmateriaal 15 jaar worden bewaard op de onderzoekslocatie. In het toestemmingformulier geef je aan of je dit goed vindt. Geef je hiervoor geen toestemming? Dan kan je nog steeds meedoen met dit onderzoek. Je krijgt dezelfde zorg.

Voor verdere uitgebreide informatie over dit onderwerp verwijzen we je naar de informatiebrief over behandeling volgens protocol SIOP-RTSG-Umbrella, die je eerder hebt gekregen. Je kan ons altijd om een nieuw exemplaar van deze informatiebrief vragen.

Registratie van het onderzoek
Informatie over dit onderzoek is ook opgenomen in een overzicht van medisch-wetenschappelijke onderzoeken, namelijk https://www.trialregister.nl/trials. Daarin zijn geen gegevens opgenomen die naar jou herleidbaar zijn. Na het onderzoek kan de website een samenvatting van de resultaten van dit onderzoek tonen. Je vindt dit onderzoek onder de naam FIURTT-Studie.

11. Verzekering

Voor iedereen die meedoet aan dit onderzoek is een verzekering afgesloten. De verzekering dekt schade door het onderzoek. Niet alle schade is gedekt. In **bijlage B** vindt u meer informatie over de verzekering en de uitzonderingen. Daar staat ook aan wie u schade kunt melden.

12. Informeren van de huisarts en/of behandelend specialist

Wij zullen jouw huisarts niet standaard apart informeren dat je meedoet aan dit onderzoek. De behandelend kinderoncoloog en kinderchirurg zijn wel op de hoogte.

13. Vergoeding voor meedoen

Het onderzoek kost jou of jouw ouders niets. Je wordt niet betaald voor het meedoen aan dit onderzoek.

14. Heb je vragen?

Bij vragen kan je contact opnemen met de onderzoeker of het onderzoeksteam. Voor onafhankelijk advies over meedoen aan dit onderzoek kan je terecht bij de onafhankelijke arts. Alle gegevens vind je in **bijlage A: Contactgegevens.**

15. Ondertekening toestemmingsformulier

Als je na zorgvuldige overweging besluit dat je deel gaat nemen aan dit wetenschappelijk onderzoek, dan vragen we je om samen met de dokter het toestemmingsformulier te ondertekenen en er een datum op te zetten. Je krijgt een kopie van deze toestemmingsverklaring na het ondertekenen.

Met vriendelijke groet,

Prof. dr. M.M. van den Heuvel-Eibrink
Kinderarts-oncoloog, hoofdonderzoeker

Prof. dr. M.H.W. Wijnen
Kinderchirurg, hoogleraar kinderoncologische chirurgie

Prof. dr. J.H.J. Hoeijmakers
Moleculair bioloog, moleculair geneticus

Drs. C.A.J. Oudmaijer
Coördinerende arts-onderzoeker

Officiële titel: Kortdurend vasten voor kinderen met unilaterale niertumoren ter vermindering van toxiciteit en bevorderen postoperatief herstel

Ik ben gevraagd om toestemming te geven voor deelname aan dit medisch-wetenschappelijke onderzoek.

Naam:______________________________Geboortedatum__/____/_____

Ik heb de informatiebrief gelezen. Ik kon vragen stellen. Mijn vragen zijn voldoende beantwoord. Ik had genoeg tijd om te beslissen of ik meedoe.

Ik weet dat meedoen vrijwillig is. Ook weet ik dat ik op ieder moment kan beslissen om toch niet mee te doen of te stoppen met het onderzoek. Daarvoor hoef ik geen reden te geven.

Ik geef toestemming voor het informeren van mijn huisarts en/of specialist(en) die mij behandelen dat ik meedoe aan dit onderzoek indien dit nodig is.

Ik geef toestemming voor het opvragen van informatie bij mijn specialist over mijn huidige behandeling en uitslagen.

Ik weet dat sommige mensen mijn gegevens kunnen inzien. Die mensen staan vermeld in deze informatiebrief.

Ik geef toestemming voor het verzamelen en gebruiken van mijn gegevens en bloed, op de manier en voor de doelen die in de informatiebrief staan.

Ik geef toestemming om mijn gegevens nog 15 jaar na dit onderzoek te bewaren.

Ik geef
□ **wel**
□ **geen**
Toestemming om mijn lichaamsmateriaal nog 15 jaar na dit onderzoek te bewaren. Mogelijk kan dit later nog voor meer onderzoek worden gebruikt, zoals in de informatiebrief staat.

Ik wil
□ **wel**
□ **niet**
Geïnformeerd worden over de resultaten van het onderzoek.

Ik geef
□ **wel**
□ **geen**
Toestemming om na dit onderzoek opnieuw benaderd te worden voor een vervolgonderzoek.

Na het ondertekenen van dit toestemmingsformulier zal ik een kopie van de ondertekenpagina ontvangen.

Ik wil meedoen aan dit onderzoek.

Naam:

Handtekening: Datum:___/___/ ___

Ik verklaar dat ik deze proefpersoon volledig heb geïnformeerd over het genoemde onderzoek.
Als er tijdens het onderzoek informatie bekend wordt die de toestemming van de proefpersoon zou kunnen beïnvloeden, dan breng ik hem/haar daarvan tijdig op de hoogte.

Naam arts:……………………………………….

Handtekening: Datum:____ /____/ _____

Aanvullende informatie is gegeven door (indien van toepassing):

Naam:

Functie:

Handtekening: Datum:____ /____/ _____

De proefpersoon krijgt een volledige informatiebrief mee, samen met een kopie van het getekende toestemmingsformulier.

Bijlagen

- Online pagina Medisch-wetenschappelijk onderzoek: https://www.rijksoverheid.nl/onderwerpen/medisch-wetenschappelijk-onderzoek
- Folder VKN (Vereniging Kinderkanker Nederland) over klinisch onderzoek.
- Bijlage A: Contactgegevens voor het Prinses Máxima Centrum
- Bijlage B: informatie over de verzekering
- Bijlage C: Tabel onderzoekshandelingen
- Bijlage D: Flyer / Patiëntenkaart FIURTT-Studie

Bijlage A: Contactgegevens voor het Prinses Máxima Centrum

Hoofdonderzoekers:
Prof. dr. M.M. van den Heuvel-Eibrink, kinderarts-oncoloog
Heidelberglaan 25, 3584 CS Utrecht
Tel: te bereiken via secretariaat Prinses Máxima Centrum, tel 06 50 00 65 70

Prof. dr. M.H.W. Wijnen, Kinderchirurg, hoogleraar kinderoncologische chirurgie
Heidelberglaan 25, 3584 CS Utrecht
Tel: te bereiken via secretariaat Prinses Máxima Centrum, tel 06 50 00 65 70

Onderzoekers:
Drs. C.A.J. Oudmaijer, arts-onderzoeker
Heidelberglaan 25, 3585 CS Utrecht
Email: c.a.j.oudmaijer@prinsesmaximacentrum.nl
Tel: 06-50173127

Researchverpleegkundigen:
Tel: 06-25710524
Email: researchnurses@prinsesmaximacentrum.nl

Onafhankelijk arts:
Als je twijfelt over deelname aan het onderzoek, dan kan je een onafhankelijke arts raadplegen die zelf niet bij het onderzoek is betrokken maar wel deskundig is op dit gebied.

Dr. M. Bierings, kinderarts-oncoloog
Heidelberglaan 25, Utrecht
Tel: te bereiken via secretariaat Prinses Máxima Centrum, 06 5000 6115

Ook als je voor of tijdens het onderzoek vragen hebt die je liever niet aan de onderzoekers stelt dan kan je contact opnemen met de onafhankelijke arts.

Klachtenbemiddeling
Als je een klacht wilt indienen, dan kunt je hiervoor contact opnemen met de ombudsvrouw van het Prinses Máxima Centrum. Zij probeert samen met jou, je ouders en de betrokkenen tot een oplossing te komen.

De ombudsvrouw is dagelijks bereikbaar op het telefoonnummer 0650006416 of via de mail: ombudsvrouw@prinsesmaximacentrum.nl.

Functionaris Gegevensbescherming
Bij vragen of klachten over de verwerking van je persoonsgegevens kan je contact opnemen met de Functionaris voor de Gegevensbescherming van het Prinses Máxima Centrum (fg@prinsesmaximacentrum.nl).
Meer informatie over je rechten bij de verwerking van je persoonsgegevens kan je vinden op de website van de Autoriteit Persoonsgegevens (https://autoriteitpersoonsgegevens.nl/nl/onderwerpen/avg-nieuwe-europese-privacywetgeving/controle-over-je-data)

Bijlage B: informatie over de verzekering

Voor iedereen die meedoet aan dit onderzoek, heeft het Prinses Máxima Centrum een verzekering afgesloten. De verzekering dekt schade door deelname aan het onderzoek. Dit geldt voor schade tijdens het onderzoek of binnen vier jaar na het einde van de deelname aan het onderzoek. Schade moet u binnen die vier jaar aan de verzekeraar hebben gemeld.

De verzekering dekt niet alle schade. Onderaan deze tekst staat in het kort welke schade niet wordt gedekt. Deze bepalingen staan in het 'Besluit verplichte verzekering bij medisch-wetenschappelijk onderzoek met mensen 2015'. Dit besluit staat in de Wettenbank van de overheid (https://wetten.overheid.nl).

Bij schade kunt u direct contact leggen met de verzekeraar.

De verzekeraar van het onderzoek is:	
Naam:	CNA Insurance Company Ltd
Adres:	Polarisavenue 140, 2132 JX Hoofddorp
Telefoonnummer:	+31 (0)23 303 6000
Polisnummer:	10211864
Contactpersoon:	Mw. Esther van Herk

De verzekering biedt een dekking van € 650.000 per proefpersoon en € 5.000.000 voor het hele onderzoek en € 7.500.000 voor alle onderzoeken van dezelfde opdrachtgever.

De verzekering dekt de volgende schade **niet**:

- schade door een risico waarover u in de schriftelijke informatie bent ingelicht. Dit geldt niet als het risico zich ernstiger voordoet dan was voorzien of als het risico heel onwaarschijnlijk was;
- schade aan de gezondheid van uw kind die ook zou zijn ontstaan als uw kind niet aan het onderzoek had meegedaan;
- schade door het niet (volledig) opvolgen van aanwijzingen of instructies;
- schade aan de nakomelingen van uw kind, als gevolg van een negatief effect van het onderzoek op uw kind of op de nakomelingen van uw kind;
- schade door een bestaande behandelmethode bij onderzoek naar bestaande behandelmethoden.

Bijlage C: Tabel onderzoekshandelingen

Tijdstip	Onderzoekshandelingen
**14 Dagen voor de operatie**	- Je behandelend arts bespreekt met je de mogelijkheid om deel te nemen aan het onderzoek.<br>- Indien je eventueel wilt deelnemen, krijg je een informatiegesprek met de arts-onderzoeker, je krijgt uitgebreide voorlichting t.a.v. wat het onderzoek inhoudt en gelegenheid voor het stellen van al je vragen.<br>- Aansluitend krijg je bedenktijd van één week.
**7 Dagen voor de operatie**	- Je hebt nogmaals een gesprek met de arts-onderzoeker over deelname aan het onderzoek.<br>- Indien je toestemming geeft voor deelname, ondertekenen jij en de arts-onderzoeker het toestemmingsformulier.<br>- Aansluitend wordt de loting t.a.v. de onderzoeksgroep verricht, je krijgt de uitslag gelijk te horen.<br>- Je krijgt een aanvullende afspraak voor beoordeling door de kinderfysiotherapeut.<br>- Tijdens deze aanvullende afspraak krijg je ook instructies t.a.v. de Accelerometer.<br>- Je start met het dragen van de Accelerometer volgens de instructies.
**1 Dag voor de operatie**	- Je wordt rond 14:00 opgenomen in het Prinses Máxima Centrum.<br>- De arts-onderzoeker loopt langs op de afdeling voor resterende vragen en instructies.<br>- Als je bent ingeloot in de vasten-groep, start je met vasten om 14:00.<br>- Jij en jouw ouders vullen een vragenlijst via het KLIK-Portaal in over hoe je je voelt tijdens de vastenperiode.
**Operatiedag**	- De arts-onderzoeker loopt langs op de afdeling ter controle.<br>- Voor het onderzoek worden er een bloedsuikercontrole en extra bloedonderzoek afgenomen.<br>- De operatie start rond 08:00, de operatie is niet anders door deelname aan het onderzoek.<br>- Het weefselonderzoek vindt pas plaats nadat de operatie klaar is.
**1 Dag na de operatie**	- In het kader van het onderzoek worden extra metingen verricht in de urine.
**2 Dagen na de operatie**	- In het kader van het onderzoek worden extra metingen verricht in de urine.
**3-6 Dagen na de operatie**	- Afhankelijk van het herstel mag je eventueel in deze periode met ontslag.<br>- Indien je met ontslag gaat, vullen jij en je ouders een vragenlijst in via het KLIK-Portaal over hoe je je voelde tijdens de afgelopen 2-3 dagen.
**+/- 8 Dagen na de operatie**	- Je hebt een reguliere poliklinische afspraak bij je behandelend arts met standaard bloedonderzoek.<br>- Jij en je ouders vullen een vragenlijst via het KLIK-Portaal in over hoe je je voelde tijdens de afgelopen 3 dagen.
**+/- 28 Dagen na de operatie**	- Je hebt een reguliere poliklinische afspraak bij je behandelend arts met standaard bloedonderzoek, er wordt i.v.m. het onderzoek extra urine afgenomen.<br>- Je krijgt een aanvullende afspraak voor een beoordeling door de kinderfysiotherapeut.<br>- Tijdens deze aanvullende afspraak lever je ook de Accelerometer in.<br>- Je krijgt een aanvullende afspraak bij de arts-onderzoeker voor het afsluiten van het onderzoek.
