# Supplementary material for: Fasting Intervention for Children With Unilateral Renal Tumors to Reduce Toxicity
Source: Front Pediatr. 2022 Jan 27;10:828615. doi: 10.3389/fped.2022.828615 (PMC8829466; doi:10.3389/fped.2022.828615)
Supplement: Supplementary file 5 [file Data_Sheet_3.pdf]

Informatiebrief met toestemmingsformulier voor ouder(s)/voogd voor deelname aan medisch wetenschappelijk onderzoek: de FIURTT Studie

Vasten voor de operatie en het herstel na een operatie voor een niertumor
Officiële titel: Kortdurend vasten voor kinderen met unilaterale niertumoren ter vermindering van toxiciteit en bevorderen postoperatief herstel

Inleiding

Beste ouder(s) – voogd,

Wij vragen u om uw kind mee te laten doen aan een medisch-wetenschappelijk onderzoek. Meedoen is vrijwillig. Om mee te doen is wel uw schriftelijke toestemming nodig.

Voordat u beslist of u uw kind wilt laten meedoen aan dit onderzoek, krijgt u uitleg over wat het onderzoek inhoudt. Lees deze informatie rustig door en vraag de arts of onderzoeker uitleg als u vragen heeft. U kunt ook de onafhankelijk deskundige, die aan het eind van deze brief genoemd wordt, om aanvullende informatie vragen. U kunt er ook over praten met uw partner, vrienden of familie.

Verdere informatie over meedoen aan zo'n onderzoek staat op de online pagina 'Medisch-wetenschappelijk onderzoek'. Deze pagina kunt u vinden via https://www.rijksoverheid.nl/onderwerpen/medisch-wetenschappelijk-onderzoek. Er is ook een folder van de VKN (Vereniging Kinderkanker Nederland) over klinisch onderzoek. Deze folder zit in de dagboekagenda die u aan het begin van de behandeling krijgt.

Heeft u of uw kind na het lezen van de informatie nog vragen? Dan kunt u terecht bij de behandelend arts of de researchverpleegkundige. In **Bijlage A** vindt u de contactgegevens.

U en uw kind (boven 12 jaar) beslissen samen of uw kind meedoet of niet.

1. Algemene informatie

U ontvangt deze brief omdat uw kind onder behandeling is op de afdeling kinderoncologie voor een niertumor. Tijdens deze behandeling krijgt uw kind eerst 4 weken lang 1 dag in de week chemotherapie en aansluitend zal er een operatie plaatsvinden in de 5^{e} of 6^{e} week. Wij willen onderzoeken hoe we uw kind zo goed mogelijk kunnen laten herstellen na de operatie.

Dit onderzoek is opgezet en wordt verricht door het Prinses Máxima Centrum voor Kinderoncologie. Het Prinses Máxima Centrum voor Kinderoncologie vergoedt de kosten van dit onderzoek. Voor dit onderzoek zijn ongeveer vijftig kinderen met een niertumor in de leeftijd van 6 maanden tot 19 jaar nodig.

De medisch-ethische toetsingscommissie Utrecht heeft dit onderzoek goedgekeurd. Algemene informatie over de toetsing van onderzoek vindt u op de online pagina 'Medisch-wetenschappelijk onderzoek'.

2. Doel van het onderzoek

Het doel van dit onderzoek is om te onderzoeken of vasten voor een operatie in verband met een niertumor ervoor zorgt dat uw kind na deze operatie sneller en beter herstelt. Normaal moet uw kind ook nuchter zijn voor een operatie, maar vasten houdt in dat uw kind langer (een aantal uur) voor zijn/haar operatie niet mag eten. Hoeveel uren hangt af van de leeftijd. Wij willen onderzoeken of, als uw kind een korte periode niet eet, het lichaam zich in de "beschermstand" zet. Deze "beschermstand" zorgt er mogelijk voor dat uw kind beter tegen de lichamelijke stress (oftewel schade) van de operatie kan. Het vasten zou zodoende ervoor kunnen zorgen dat uw kind sneller herstelt na de operatie en dat er minder schade is in de nier die uw kind blijft houden. Dit willen we onderzoeken bij deze ziekte en de bijbehorende operatie. Afhankelijk van de resultaten van het onderzoek willen we het vervolgens toepassen in de dagelijkse zorg.

3. Achtergrond van het onderzoek

Verschillende onderzoeken hebben laten zien dat dieren langer leven als er minder calorieën in de voeding zit dan normaal. Dit komt gedeeltelijk door een betere weerstand tegen oxidatieve stress, weerstand die het lichaam zelf opbouwt als er minder calorieën in de voeding zitten. Oxidatieve stress is een soort schade die ontstaat in situaties die schadelijk zijn voor het lichaam zoals warmte, UV-licht en roken. Oxidatieve stress ontstaat ook tijdens een operatie en kan nadelige invloed hebben op het herstel na de operatie.

Eerder onderzoek met nierdonoren die een nier hebben afgestaan en patiënten die een maagverkleining hebben ondergaan lieten zien dat een dieet of vasten voor de operatie goed werd verdragen zonder een hoger risico op complicaties na de operatie. Ook werd er een mogelijk effect op beter herstel bij de donor en de ontvanger van de nier gezien. Nu willen we onderzoeken of dit gunstige effect ook aanwezig is bij kinderen die worden geopereerd aan een niertumor.

4. Wat meedoen inhoudt

Als uw kind meedoet met dit onderzoek, zal het onderzoek aanvullend aan de standaardbehandeling voor de niertumor plaatsvinden. Het eerste deel van de standaardbehandeling voor een niertumor neemt ongeveer 6 weken in beslag. Dit bestaat uit 1 maand chemotherapie (4 keer een cyclus van 1 week), een operatie en het herstel na de operatie.

Het vasten vindt plaats vanaf 1 dag voor de operatie en dit duurt tot en met de operatie. Na de operatie mag uw kind weer eten afhankelijk van hoe de operatie gegaan is.

Geschiktheidsonderzoek
Op basis van medische gegevens van uw kind zal gekeken worden, in samenspraak met zijn/haar behandelend arts, of er bezwaren zijn voor het meedoen aan het onderzoek. Hiervoor zijn geen extra onderzoeken nodig.

Het onderzoek
Wanneer u (en uw kind) toestemming geeft en meedoet aan dit onderzoek, komt uw kind via willekeurige loting in één van twee onderzoeksgroepen. Dit betekent dat uw kind 50% kans heeft om in de vastengroep geloot te worden.

Groep 1:
De standaardbehandeling: uw kind wordt 6 uur (vanaf 02:00) voor de operatie nuchter gehouden. Nuchter zijn betekent geen vast eten. Uw kind mag tot vlak voor de operatie nog wel heldere suikerhoudende dranken drinken.

Groep 2:
De behandeling die we onderzoeken: uw kind wordt 10, 14 of 18 uur (*afhankelijk van de leeftijd) voor de operatie nuchter gehouden. Uw kind mag niet eten. Tot vlak voor de operatie mag er wel suikervrije limonade, water of thee gedronken worden, als er maar geen calorieën in zitten (dus geen suiker in de thee).

*Leeftijdsgroep 1:	0,5 tot 2 jaar oud (6-24 maanden):	10 uur vasten, vanaf 22:00
*Leeftijdsgroep 2:	2 tot 7 jaar oud:	14 uur vasten, vanaf 18:00
*Leeftijdsgroep 3:	7 tot 18 jaar oud:	18 uur vasten, vanaf 14:00

Het vasten start pas als uw kind is opgenomen in het ziekenhuis, zie ook bijlage B.

Wat is er meer of anders dan de gebruikelijke zorg of behandeling?
Normaal komt u ongeveer 1 keer per week bij de behandelend arts en duurt een controle een half uur. De bezoeken die bij dit onderzoek horen, vinden plaats op het moment van de normale controles en daardoor kunnen de controles 15-20 minuten langer duren. Als uw kind ingeloot wordt voor de behandeling die we onderzoeken, dan mag uw kind voor de operatie voor een aantal uren langer durende periode niet eten. Zie de volgende alinea voor wat er precies extra gebeurt in verband met het onderzoek.

Bezoeken en metingen
Voor het onderzoek zijn geen extra afspraken nodig in het ziekenhuis. U en uw kind komen op de reguliere afspraakmomenten langs. Wel is het mogelijk dat de afspraak iets langer duurt als u meedoet aan het onderzoek, dit is maximaal 15 tot 20 minuten langer dan wanneer uw kind niet meedoet.

In het eerste deel van de behandeling komt uw kind wekelijks naar het ziekenhuis voor de medicijnbehandeling. In de derde week van de medicijnbehandeling gaat dit onderzoek lopen en gaan we extra gegevens verzamelen.

Dit zijn:

- Voor de operatie, tijdens de ziekenhuisopname en 2-3 dagen na de opname willen we door middel van een klein apparaatje meten hoeveel uw kind beweegt/hoe actief uw kind is. Dit kleine apparaatje zit op een soort riem die om de heup van uw kind zit. Dit apparaatje wordt al in het ziekenhuis gebruikt. In verband met dit onderzoek krijgt u dit apparaatje voor de operatie alvast mee naar huis om samen met uw kind te oefenen met het dragen en om te kijken hoe actief uw kind is voor de operatie. Kinderen hebben over het algemeen weinig last van het dragen van het apparaatje, in principe kunnen ze alles doen wat ze normaal ook zouden doen. Onze kinderfysiotherapeut zal u en uw kind helpen met hoe het apparaatje werkt en hoe u het moet gebruiken.
- Voor en na de operatie krijgt uw kind een aanvullende afspraak bij de kinderfysiotherapeut. Tijdens deze afspraak van ongeveer 20 minuten zullen verschillende metingen verricht worden t.a.v. de lichamelijke conditie van uw kind.
- Voor de operatie wordt het bloedsuikergehalte gecontroleerd door middel van een vingerprik. Dit is een kleine prik waar uw kind weinig last van heeft.
- Voor de nieroperatie krijgt uw kind altijd een infuus, uw kind krijgt hier medicijnen door, maar ook wordt er meermaals bloed via afgenomen. We willen hier twee keer één extra buisje bloed uit afnemen tijdens een normaal afnamemoment. We nemen bloed af uit de infuuslijn om te meten hoe het lichaam reageert op het vasten. Uw kind krijgt dus geen extra prik.
- Rondom de operatie krijgt uw kind altijd een slangetje in zijn/haar urinebuis (een katheter). Daar nemen we een aantal dagen urine uit af voor onderzoek.
  Ook vragen we uw kind om urine mee te nemen als hij/zij na 4 weken op de polikliniek terugkomt.
- U en uw kind krijgen 3x een vragenlijst over hoe het met uw kind gaat. De vragenlijst kost u per keer ongeveer 10-15 minuten, de vragenlijst wordt digitaal ingevuld via het KLIK-Portaal van het Prinses Máxima Centrum. Dit doen we om na te gaan hoe belastend het vasten voor u en uw kind is.
- Er wordt door een patholoog (een arts die gespecialiseerd is in het onderzoeken van lichaamsweefsel en cellen) een stukje afgenomen van de niertumor tijdens het weefselonderzoek na de operatie. Dit weefsel wordt gebruikt om te onderzoeken hoe de cellen hebben gereageerd op het vasten. Hier ondervindt uw kind geen last van.

Er is ook informatie die we in verband met het onderzoek gebruiken, maar die we al verzamelen als <u>onderdeel van de standaardbehandeling:</u>

- Tijdens iedere afspraak zullen we lengte en gewicht meten, ook als uw kind in het ziekenhuis is.
- Alle andere bloedafnames gaan door zoals dat normaal ook gaat.
- We houden de urineproductie bij zoals dat normaal ook gaat.

5. Wat wordt er van u en uw kind verwacht

Om het onderzoek goed te laten verlopen en voor de veiligheid van uw kind, is het belangrijk dat u zich aan de volgende afspraken houdt: de afspraken zijn dat u en uw kind:

- Het vastendieet volgen volgens voorschrift/uitleg tijdens de opname.
- De vragenlijsten invullen.
- De activiteitenmeter gebruiken zoals dat is uitgelegd.
- Niet ook aan een ander medisch-wetenschappelijk onderzoek meedoet zonder medeweten van de onderzoeker.
- De afspraken voor bezoeken zo goed mogelijk nakomen.

Het is belangrijk dat u contact opneemt met de onderzoeker of researchverpleegkundige:

- Voordat uw kind andere geneesmiddelen gaat gebruiken. Ook als dat homeopathische geneesmiddelen, natuurgeneesmiddelen, vitaminen en/of geneesmiddelen van de drogist zijn.
- Als uw kind in een ziekenhuis wordt opgenomen of behandeld.
- Als uw kind plotseling gezondheidsklachten krijgt.
- Als uw kind niet meer wilt meedoen aan het onderzoek.
- Als uw contactgegevens wijzigen.

U krijgt een kaartje met telefoonnummers en informatie over het onderzoek. Wij vragen u om dit kaartje mee te nemen bij elk ziekenhuisbezoek.

6. Mogelijke complicaties en eventuele nadelige effecten

Het (langer) vasten in het kader van het onderzoek kan nadelige effecten geven. Uw kind kan tijdens het dieet mogelijk een hongergevoel ervaren of licht draaierig worden. Is dit het geval, dan kunt u of uw kind dit aangeven bij de verpleegkundige en/of afdelingsarts. Daarnaast is het vasten een verandering in het dagelijks leven en kan het zodoende gepaard gaan met stress en/of onwennigheid. In zeldzame gevallen kan uw kind een te laag bloedsuikergehalte krijgen. Wij schatten echter in dat de kans hierop klein is, gezien de gekozen vastentijden. Het bloedsuikergehalte zullen we tijdens het vasten zekerheidshalve controleren. Dit kunnen we goed behandelen met een glucose infuus.

7. Mogelijke voor- en nadelen

Het is belangrijk dat u de mogelijke voor- en nadelen goed afweegt voordat u besluit mee te doen.

Het onderzoek kijkt naar de effecten van een vastendieet op het herstel na een operatie. Eerdere onderzoeken naar dit onderwerp lieten geen nadelige effecten maar juist enkele voordelige effecten zien. Deze vastentherapie voor een operatie voor een niertumor kan een gunstig effect geven, maar zeker is dat niet. We verwachten dat dit onderzoek in de toekomst bijdraagt aan beter herstel na een operatie. Uw kind draagt bij aan meer kennis over de behandeling van niertumoren bij andere kinderen/jongeren.

Als uw kind deelneemt aan het onderzoek kunt u mogelijk neveneffecten verwachten van het vasten. Hierover staat meer onder punt 6. Voor het onderzoek wordt niet een extra bloedafnamemoment verricht, maar er wordt wel tweemaal één extra buisje bloed afgenomen tijdens een standaard bloedafnamemoment. Uw kind ondervindt hier geen last van. Indien uw kind ingeloot wordt voor de behandeling die we onderzoeken, dan mag uw kind voor de operatie gedurende een langere periode niet eten. Ook zal uw kind eenmaal wat extra urine moeten inleveren. Het invullen van de vragenlijsten kost u in totaal ongeveer 3 keer 10-15 minuten in de loop van 2 maanden.

Deelname aan het onderzoek betekent dus ook:

- Dat u tijd kwijt bent aan het invullen van vragenlijsten.
- Dat uw kind thuis een aantal dagen een klein apparaatje moet dragen.
- Dat u twee extra afspraken krijgt bij de kinderfysiotherapie.
- Dat u afspraken heeft waaraan u zich moet houden.
- Dat uw kind eenmalig extra urine en tweemaal één extra buisje bloed moet inleveren voor extra onderzoek.
- Dat wij extra weefselonderzoek verrichten.

Al deze zaken zijn hiervoor onder punt 4, 5 en 6 beschreven.

8. Verzet van uw kind

Het kan zijn dat uw kind zich tijdens het onderzoek verzet (niet meewerkt). De onderzoeker moet het onderzoek dan direct stoppen. Het is moeilijk om precies te omschrijven wat verzet is. Voor de start van het onderzoek wordt met u overlegd wat als verzet wordt gezien.

Er is in het Prinses Máxima Centrum een team van gespecialiseerde medewerkers (zoals een arts, psycholoog, gespecialiseerde verpleegkundige) beschikbaar om u en uw kind tijdens het onderzoek zo goed mogelijk te begeleiden. Het behandelteam ziet nauwlettend toe op de belasting die deelname aan een onderzoeksprotocol voor uw kind met zich meebrengt. We werken daarom ook zoveel mogelijk volgens landelijke afspraken zoals die door de Nederlandse Vereniging voor Kindergeneeskunde (NVK) zijn vastgelegd ter bescherming van minderjarige onderzoeksdeelnemers. Voor meer informatie hierover verwijzen we u naar de website www.ccmo.nl onder “wet en regelgeving” gedragscode verzet: minderjarigen.

9. Als u niet wilt dat uw kind meedoet of als u wilt dat uw kind stopt met het onderzoek

U beslist zelf of uw kind meedoet aan het onderzoek. Deelname is vrijwillig. Als u niet wilt dat uw kind meedoet, wordt uw kind op de gebruikelijke manier behandeld voor de niertumor. De standaardbehandeling is chemotherapie voor 4 weken met aansluitend een operatie met de gebruikelijke periode dat uw kind nuchter moet zijn voor de operatie. De nabehandeling hangt af van de operatie en de weefseluitslag en is geen onderdeel van dit onderzoek.

Als uw kind meedoet, kunt u zich altijd bedenken en uw kind toch laten stoppen, ook tijdens het onderzoek. De arts zal dan met u en uw kind bespreken hoe de gewone behandeling en vervolgafspraken gepland worden. U hoeft niet te zeggen waarom u stopt. Wel moet u dit direct melden aan de behandelend arts. De gegevens die tot dat moment zijn verzameld, worden gebruikt voor het onderzoek. Als u wilt, kan verzameld lichaamsmateriaal worden vernietigd.

Als er nieuwe informatie over het onderzoek beschikbaar komt die belangrijk voor u en uw kind kan zijn, laat de arts dit aan u weten. U wordt dan gevraagd of uw kind mee blijft doen.

10. Einde van het onderzoek

Deelname van uw kind aan het onderzoek stopt:
- Na de poliklinische controleafspraak 4 weken na de operatie.
- Als u zelf kiest om uw kind te laten stoppen.
- Als het einde van het hele onderzoek is bereikt.
- Als de arts het beter voor uw kind vindt om te stoppen.
- Als de tumor niet te opereren is.
- Als het Prinses Máxima Centrum voor Kinderoncologie, de overheid of de beoordelende medisch-ethische toetsingscommissie, besluit om het onderzoek te stoppen.

Het hele onderzoek is afgelopen als alle deelnemers klaar zijn. Uw behandelend arts zal met u praten over de verdere medische zorg na afloop van het onderzoek. Na het verwerken van alle gegevens informeert de onderzoeker u over de belangrijkste uitkomsten van het onderzoek, als u dit wenst.

11. Gebruik en bewaren van de gegevens en lichaamsmaterialen

Voor dit onderzoek worden persoonsgegevens en lichaamsmateriaal van uw kind verzameld, gebruikt en bewaard. Het gaat om gegevens zoals naam, adres, geboortedatum en om gegevens over de gezondheid van uw kind. Voor dit onderzoek is nierweefsel (tumorweefsel & normaal nierweefsel) nodig. Het verzamelen, gebruiken en bewaren van de gegevens en lichaamsmateriaal van uw kind, gedurende het onderzoek, is nodig om de vragen die in dit onderzoek worden gesteld te kunnen beantwoorden en de resultaten te kunnen publiceren. Het lichaamsmateriaal zal voor analyse opgeslagen worden op de researchafdeling en het laboratorium van het Prinses Máxima Centrum voor Kinderoncologie. Wij vragen voor het gebruik van de gegevens en lichaamsmateriaal van uw kind uw toestemming. Als u dat niet wilt, kan uw kind niet deelnemen aan dit onderzoek.

Het lichaamsmateriaal kan na afloop van dit onderzoek ook nog van belang zijn voor ander wetenschappelijk onderzoek op het gebied van het vasten-dieet en operaties. Daarvoor zullen de gegevens en lichaamsmateriaal van uw kind 15 jaar worden bewaard op de onderzoekslocatie. In het toestemmingformulier geeft u aan of u dit goed vindt. Geeft u hiervoor geen toestemming? Dan kan uw kind nog steeds meedoen met dit onderzoek. Uw kind krijgt dezelfde zorg.

Voor verdere uitgebreide informatie over dit onderwerp verwijzen we u naar de informatiebrief over behandeling volgens protocol SIOP-RTSG-Umbrella, die u eerder heeft gekregen. U kunt ons altijd om een nieuw exemplaar van deze informatiebrief vragen.

Registratie van het onderzoek
Informatie over dit onderzoek is ook opgenomen in een overzicht van medisch-wetenschappelijke onderzoeken, namelijk https://www.trialregister.nl/trials. Daarin zijn geen gegevens opgenomen die naar uw kind herleidbaar zijn. Na het onderzoek kan de website een samenvatting van de resultaten van dit onderzoek tonen. U vindt dit onderzoek onder de naam FIURTT-Studie.

12. Verzekering

Voor iedereen die meedoet aan dit onderzoek is een verzekering afgesloten. De verzekering dekt schade door het onderzoek. Niet alle schade is gedekt. In **bijlage B** vindt u meer informatie over de verzekering en de uitzonderingen. Daar staat ook aan wie u schade kunt melden.

13. Informeren van de huisarts en/of behandelend specialist

Wij zullen uw huisarts niet standaard apart informeren dat uw kind meedoet aan dit onderzoek. De behandelend kinderoncoloog en kinderchirurg zijn wel op de hoogte.

14. Vergoeding voor meedoen

Het onderzoek kost u of uw kind niets. U of uw kind wordt niet betaald voor het meedoen aan dit onderzoek.

15. Heeft u vragen?

Bij vragen kunt u contact opnemen met de onderzoeker of het onderzoeksteam. Voor onafhankelijk advies over meedoen aan dit onderzoek kunt u terecht bij de onafhankelijke arts. Alle gegevens vindt u in **bijlage A: Contactgegevens.**

16. Ondertekening toestemmingsformulier

Wanneer u voldoende bedenktijd heeft gehad, wordt u gevraagd te beslissen over deelname aan dit onderzoek. Als u toestemming geeft, zullen wij u vragen deze op de bijbehorende toestemmingsverklaring schriftelijk te bevestigen. Door uw schriftelijke toestemming geeft u aan dat u de informatie heeft begrepen en instemt met deelname van uw kind aan het onderzoek.

Zowel uzelf als de onderzoeker of de behandelend arts ontvangen een getekende versie van deze toestemmingsverklaring.

Met vriendelijke groet,

Prof. dr. M.M. van den Heuvel-Eibrink
Kinderarts-oncoloog, hoofdonderzoeker

Prof. dr. M.H.W. Wijnen
Kinderchirurg, hoogleraar kinderoncologische chirurgie

Prof. dr. J.H.J. Hoeijmakers
Moleculair bioloog, moleculair geneticus

Drs. C.A.J. Oudmaijer
Coördinerende arts-onderzoeker

Toestemmingsformulier ouders of voogd

Officiële titel: Kortdurend vasten voor kinderen met unilaterale niertumoren ter vermindering van toxiciteit en bevorderen postoperatief herstel

Ik ben gevraagd om toestemming te geven, voor deelname van mijn kind aan dit medisch-wetenschappelijke onderzoek:

Naam kind:______________________________Geboortedatum__/____/_____

Ik heb de informatiebrief voor mijn kind gelezen. Ik kon aanvullende vragen stellen. Mijn vragen zijn genoeg beantwoord. Ik had genoeg tijd om te beslissen of ik wil dat mijn kind meedoet.

Ik weet dat meedoen vrijwillig is. Ook weet ik dat ik op ieder moment kan beslissen dat mijn kind toch niet meedoet. Daarvoor hoef ik geen reden te geven.

Ik geef toestemming voor het informeren van de huisarts en/of specialist(en) die mijn kind behandelen dat mijn kind meedoet aan dit onderzoek indien dit nodig is.

Ik weet dat voor de controle van het onderzoek sommige mensen toegang tot alle gegevens van mijn kind kunnen krijgen. Die mensen staan vermeld in deze informatiebrief. Ik geef toestemming voor die inzage door deze personen.

Ik geef toestemming voor het verzamelen en gebruiken van de gegevens, bloedmonsters en lichaamsmateriaal van mijn kind voor de beantwoording van de onderzoeksvraag in dit onderzoek.

Ik geef toestemming om de onderzoeksgegevens van mijn kind 15 jaar na afloop van dit onderzoek te bewaren.

Ik geef
□ **wel**
□ **geen**
Toestemming om het lichaamsmateriaal van mijn kind nog 15 jaar na dit onderzoek te bewaren. Mogelijk kan dit later nog voor meer onderzoek worden gebruikt, zoals in de informatiebrief staat.

Ik wil
□ **wel**
□ **niet**
Geïnformeerd worden over de resultaten van het onderzoek.

Ik geef
□ **wel**
□ **geen**
Toestemming om mijn kind na dit onderzoek opnieuw te benaderen voor een vervolgonderzoek.

Na het ondertekenen van dit toestemmingsformulier zal ik een kopie van de ondertekenpagina ontvangen.

Ik ga ermee akkoord dat deze persoon/mijn kind meedoet aan dit onderzoek.

Naam ouder/voogd**……………………………….
Handtekening: Datum:____ /_____/ ____

Naam ouder/voogd**……………………………….
Handtekening: Datum:____ /____/ _____

--- ----
Ik verklaar hierbij dat ik bovengenoemde persoon/personen volledig heb geïnformeerd over het genoemde onderzoek. Als er tijdens het onderzoek informatie bekend wordt die de toestemming van de ouder of voogd zou kunnen beïnvloeden, dan breng ik hem/haar daarvan tijdig op de hoogte.

Naam arts:……………………………………….

Handtekening: Datum:____ /____/ _____

Aanvullende informatie is gegeven door (indien van toepassing):

Naam:

Functie:

Handtekening: Datum:____ /__/ _____

----

* *Doorhalen wat niet van toepassing is.*
*** Wanneer het kind jonger dan 16 jaar is, ondertekenen de ouders die het gezag uitoefenen of de voogd dit formulier. Kinderen van 12 t/m 15 jaar die zelfstandig beslissingen kunnen nemen (wilsbekwaam zijn), moeten ook het Toestemmingsformulier voor kinderen en jongeren ondertekenen.*

De ouder/voogd krijgt een volledige informatiebrief mee, samen met een getekende versie van het toestemmingsformulier.

Bijlagen
- Online pagina Medisch-wetenschappelijk onderzoek:
https://www.rijksoverheid.nl/onderwerpen/medisch-wetenschappelijk-onderzoek
- Folder VKN (Vereniging Kinderkanker Nederland) over klinisch onderzoek.
- Bijlage A: Contactgegevens voor het Prinses Máxima Centrum
- Bijlage B: informatie over de verzekering
- Bijlage C: Tabel onderzoekshandelingen
- Bijlage D: Flyer / Patiëntenkaart FIURTT-Studie

Bijlage A: Contactgegevens voor het Prinses Máxima Centrum

Hoofdonderzoekers:
Prof. dr. M.M. van den Heuvel-Eibrink, kinderarts oncoloog
Heidelberglaan 25, 3584 CS Utrecht
Tel: te bereiken via secretariaat Prinses Máxima Centrum, tel 06 50 00 65 70

Prof. dr. M.H.W. Wijnen, Kinderchirurg, hoogleraar kinderoncologische chirurgie
Heidelberglaan 25, 3584 CS Utrecht
Tel: te bereiken via secretariaat Prinses Máxima Centrum, tel 06 50 00 65 70

Onderzoekers:
Drs. C.A.J. Oudmaijer, arts-onderzoeker
Heidelberglaan 25, 3585 CS Utrecht
Email: c.a.j.oudmaijer@prinsesmaximacentrum.nl
Tel: 06-50173127

Researchverpleegkundigen:
Tel: 06-25710524
Email: researchnurses@prinsesmaximacentrum.nl

Onafhankelijk arts:
Als u twijfelt over deelname van uw kind aan het onderzoek, dan kunt u een onafhankelijke arts raadplegen die zelf niet bij het onderzoek is betrokken maar wel deskundig is op dit gebied.

Dr. M. Bierings, kinderarts-oncoloog
Heidelberglaan 25, Utrecht
Tel: te bereiken via secretariaat Prinses Máxima Centrum, 06 5000 6115

Ook als u voor of tijdens het onderzoek vragen heeft die u liever niet aan de onderzoekers stelt dan kunt u contact opnemen met de onafhankelijke arts.

Klachtenbemiddeling
Als u of uw kind een klacht wil indienen, dan kunt u hiervoor contact opnemen met de ombudsvrouw van het Prinses Máxima Centrum. Zij probeert samen met u, uw kind en de betrokkenen tot een oplossing te komen.

De ombudsvrouw is dagelijks bereikbaar op het telefoonnummer 0650006416 of via de mail: ombudsvrouw@prinsesmaximacentrum.nl.

Functionaris Gegevensbescherming
Bij vragen of klachten over de verwerking van de persoonsgegevens van uw kind kunt u contact opnemen met de Functionaris voor de Gegevensbescherming van het Prinses Máxima Centrum (fg@prinsesmaximacentrum.nl). Meer informatie over uw rechten bij de verwerking van de persoonsgegevens van uw kind kunt u vinden op de website van de Autoriteit Persoonsgegevens (https://autoriteitpersoonsgegevens.nl/nl/onderwerpen/avg-nieuwe-europese-privacywetgeving/controle-over-je-data).

Bijlage B: informatie over de verzekering

Voor iedereen die meedoet aan dit onderzoek, heeft het Prinses Máxima Centrum een verzekering afgesloten. De verzekering dekt schade door deelname aan het onderzoek. Dit geldt voor schade tijdens het onderzoek of binnen vier jaar na het einde van de deelname aan het onderzoek. Schade moet u binnen die vier jaar aan de verzekeraar hebben gemeld.

De verzekering dekt niet alle schade. Onderaan deze tekst staat in het kort welke schade niet wordt gedekt. Deze bepalingen staan in het 'Besluit verplichte verzekering bij medisch-wetenschappelijk onderzoek met mensen 2015'. Dit besluit staat in de Wettenbank van de overheid (https://wetten.overheid.nl).

Bij schade kunt u direct contact leggen met de verzekeraar.

De verzekeraar van het onderzoek is:	
Naam:	CNA Insurance Company Ltd
Adres:	Polarisavenue 140, 2132 JX Hoofddorp
Telefoonnummer:	+31 (0)23 303 6000
Polisnummer:	10211864
Contactpersoon:	Mw. Esther van Herk

De verzekering biedt een dekking van € 650.000 per proefpersoon en € 5.000.000 voor het hele onderzoek en € 7.500.000 voor alle onderzoeken van dezelfde opdrachtgever.

De verzekering dekt de volgende schade **niet**:

- schade door een risico waarover u in de schriftelijke informatie bent ingelicht. Dit geldt niet als het risico zich ernstiger voordoet dan was voorzien of als het risico heel onwaarschijnlijk was;
- schade aan de gezondheid van uw kind die ook zou zijn ontstaan als uw kind niet aan het onderzoek had meegedaan;
- schade door het niet (volledig) opvolgen van aanwijzingen of instructies;
- schade aan de nakomelingen van uw kind, als gevolg van een negatief effect van het onderzoek op uw kind of op de nakomelingen van uw kind;
- schade door een bestaande behandelmethode bij onderzoek naar bestaande behandelmethoden.

Bijlage C: Tabel onderzoekshandelingen

Tijdstip	Onderzoekshandelingen
**14 Dagen voor de operatie**	- Uw behandelend arts bespreekt met u de mogelijkheid om deel te nemen aan het onderzoek.<br>- Indien u eventueel wilt deelnemen, krijgt u een informatiegesprek met de arts-onderzoeker, u krijgt uitgebreide voorlichting t.a.v. wat het onderzoek inhoudt en gelegenheid voor het stellen van al uw vragen.<br>- Aansluitend krijgt u bedenktijd van één week.
**7 Dagen voor de operatie**	- U heeft nogmaals een gesprek met de arts-onderzoeker over deelname aan het onderzoek.<br>- Indien u toestemming geeft voor deelname, ondertekenen de ouders, de onderzoeker en eventueel uw kind het toestemmingsformulier.<br>- Aansluitend wordt de loting t.a.v. de onderzoeksgroep verricht, u krijgt de uitslag gelijk te horen.<br>- U krijgt een aanvullende afspraak voor beoordeling door de kinderfysiotherapeut.<br>- Tijdens deze aanvullende afspraak krijgt u ook instructies t.a.v. de Accelerometer.<br>- Uw kind start met het dragen van de Accelerometer volgens de instructies.
**1 Dag voor de operatie**	- Uw kind wordt rond 14:00 opgenomen in het Prinses Máxima Centrum.<br>- De arts-onderzoeker loopt langs op de afdeling voor resterende vragen en instructies.<br>- Als uw kind is ingeloot in de vasten-groep, start uw kind met vasten. De starttijd is afhankelijk van zijn/haar leeftijd (14:00, 18:00 of 22:00 uur).<br>- U vult een vragenlijst via het KLIK-Portaal in over hoe uw kind zich voelt tijdens de vastenperiode.
**Operatiedag**	- De arts-onderzoeker loopt langs op de afdeling ter controle.<br>- Voor het onderzoek worden er een bloedsuikercontrole en extra bloedonderzoek afgenomen.<br>- De operatie start rond 08:00, de operatie is niet anders door deelname aan het onderzoek.<br>- Het weefselonderzoek vindt pas plaats nadat de operatie klaar is.
**1 Dag na de operatie**	- In het kader van het onderzoek worden extra metingen verricht in de urine.
**2 Dagen na de operatie**	- In het kader van het onderzoek worden extra metingen verricht in de urine.
**3-6 Dagen na de operatie**	- Afhankelijk van het herstel mag uw kind eventueel in deze periode met ontslag.<br>- Indien uw kind met ontslag gaat, vult u een vragenlijst in via het KLIK-Portaal over hoe uw kind zich voelde tijdens de afgelopen 2-3 dagen.
**+/- 8 Dagen na de operatie**	- U vult een vragenlijst via het KLIK-Portaal in over hoe uw kind zich voelde tijdens de afgelopen 3 dagen.
**+/- 28 Dagen na de operatie**	- U heeft een reguliere poliklinische afspraak bij uw behandelend arts met standaard bloedonderzoek, er wordt i.v.m. het onderzoek extra urine afgenomen.<br>- U krijgt een aanvullende afspraak voor een beoordeling door de kinderfysiotherapeut.<br>- Tijdens deze aanvullende afspraak levert u ook de Accelerometer in.<br>- U krijgt een aanvullende afspraak bij de arts-onderzoeker voor het afsluiten van het onderzoek.
