# Supplementary material for: Fasting Intervention for Children With Unilateral Renal Tumors to Reduce Toxicity
Source: Front Pediatr. 2022 Jan 27;10:828615. doi: 10.3389/fped.2022.828615 (PMC8829466; doi:10.3389/fped.2022.828615)
Supplement: Supplementary file 6 [file Data_Sheet_4.pdf]

Informatiebrief met toestemmingsformulier voor kinderen (12 tot 16 jaar) voor deelname aan medisch wetenschappelijk onderzoek: de FIURTT Studie

---

Vasten voor de operatie en het herstel na een operatie voor een niertumor
Officiële titel: Kortdurend vasten voor kinderen met unilaterale niertumoren ter vermindering van toxiciteit en bevorderen postoperatief herstel

Beste,

Doe je mee aan een onderzoek? Hier lees je meer over het onderzoek en jouw rechten. Lees dit goed, want dan weet je waarover je kunt beslissen. Je mag rustig nadenken voordat je beslist.

Je ouders krijgen ook informatie over dit onderzoek. Je kunt samen met hen praten over het onderzoek. Zij zullen samen met jou een beslissing nemen.

Meer informatie over meedoen aan een onderzoek kun je online vinden op de pagina 'Medisch-wetenschappelijk onderzoek' via https://www.rijksoverheid.nl/onderwerpen/medisch-wetenschappelijk-onderzoek. Er is ook een folder van de VKN (Vereniging Kinderkanker Nederland) over klinisch onderzoek.

Vragen en contact

Heb je vragen? Bespreek ze met je ouders. Of stel ze aan de arts of de onderzoeksverpleegkundige. Je kunt je vragen hieronder opschrijven.

Je mag de arts of de onderzoeksverpleegkundige ook altijd bellen of mailen:

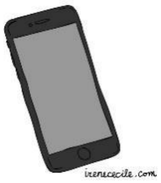

- Prof. dr. M.M. van den Heuvel-Eibrink en/of prof. dr. M.H.W. Wijnen, bereikbaar via het telefoonnummer 088 972 5206.
- De arts-onderzoeker, drs. C.A.J. (Chris) Oudmaijer, bereikbaar via het telefoonnummer 06 50173127 of via e-mail: c.a.j.oudmaijer@prinsesmaximacentrum.nl.
- De onderzoeksverpleegkundige, bereikbaar via het telefoonnummer 06 25710524 of stuur een e-mail naar researchnurses@prinsesmaximacentrum.nl.

Wil je praten over het onderzoek met een arts die er niet bij betrokken is? Bel dan met:

- Dr. M. Bierings, bereikbaar via het telefoonnummer 06 - 50006115.

Ruimte om jouw vragen op te schrijven:

Tip: neem een foto van je vragen, dan heb je ze bij je als je met de arts/onderzoeker gaat praten.

Over het onderzoek

Dit onderzoek gebeurt in het Prinses Máxima Centrum voor Kinderoncologie. Er doen ongeveer 50 kinderen en/of jongeren met dit onderzoek mee. Het onderzoek is gecontroleerd en goedgekeurd. De naam van de commissie die de beoordeling heeft gedaan is de METC Utrecht.

Waarom dit onderzoek?

Je ontvangt deze brief omdat bij jou een kwaadaardig gezwel in de nier is vastgesteld. Dit is een zwelling van je nier die behandeld moet worden met medicijnen en een operatie. De dokter heeft je verteld dat je 4 weken lang 1 dag per week naar het ziekenhuis moet komen om medicijnen te krijgen. Daarna vindt er een operatie plaats.

Het doel van dit onderzoek is om te onderzoeken of vasten voor deze operatie ervoor zorgt dat je na de operatie sneller en beter herstelt. Normaal moet je ook nuchter zijn voor een operatie, maar vasten houdt in dat je langer (een aantal uur) voor je operatie niet mag eten. Wij willen onderzoeken of, als je een korte periode niet eet, je lichaam zich in de “beschermstand” zet. Deze “beschermstand” zorgt er mogelijk voor dat je beter tegen de lichamelijke stress van de operatie kan en daardoor minder schade krijgt. Het vasten zou ervoor kunnen zorgen dat je zo goed mogelijk herstelt na de operatie en dat er minder schade is in de nier die je blijft houden. Afhankelijk van de resultaten van het onderzoek willen we het vervolgens toepassen in de dagelijkse zorg.

Achtergrond:

Verschillende onderzoeken hebben laten zien dat dieren langer leven als ze net iets minder eten dan normaal. Dit komt gedeeltelijk door een betere weerstand tegen lichamelijke stress, ook wel schade genoemd. Deze weerstand bouwt het lichaam zelf op als je een korte tijd iets minder eet. Deze schade ontstaat ook tijdens een operatie. We deden al eerder onderzoek naar het effect van vasten voor een operatie bij volwassen mensen. Daar zagen we dat het dieet en/of vasten goed ging en dat ze niet meer problemen kregen van de operatie en we zagen een iets sneller herstel.

Wat houdt het onderzoek in?

Het totale onderzoek duurt ongeveer 6 weken en vindt tegelijkertijd plaats met de standaardbehandeling die je gaat krijgen. Er zijn 2 groepen in het onderzoek. We loten van tevoren in welke groep jij terecht komt, dit betekent dat je 50% kans hebt om in de vastengroep geloot te worden. We vertellen je gelijk in welke groep jij terecht komt. Eén groep zal vanaf 18 uur voor de operatie niks meer mogen eten en de andere groep vanaf 6 uur voor de operatie. Wil je meer weten over waarom we loten? Dan kun je dit vragen aan de arts of de onderzoeksverpleegkundige.

We gaan eerst kijken of je mee kunt doen. Dit is allemaal onderdeel van jouw normale behandeling voor het gezwel in de nier, maar er zal extra gekeken worden of je mee kan doen met het onderzoek, in samenspraak met je behandelend arts. Hiervoor zijn geen extra onderzoeken nodig. Normaal kom je ongeveer 1 keer per week bij de dokter en duurt een controle een half uur. De bezoeken die bij dit onderzoek horen, vinden plaats op het moment van de normale controles en daardoor kunnen de controles 15-20 minuten langer duren.

In het eerste deel van je behandeling kom je wekelijks naar het ziekenhuis voor de medicijnbehandeling. In de derde week van de medicijnbehandeling gaat dit onderzoek pas lopen en gaan we extra dingen meten. Dit zijn:

- Voor de operatie, tijdens de ziekenhuisopname en 2-3 dagen na de opname willen we door middel van een apparaatje meten hoeveel je beweegt. Dit kleine apparaatje zit op een soort riem die om je heup zit. Dit apparaatje wordt al in het ziekenhuis gebruikt. In verband met dit onderzoek krijg je dit apparaatje voor de operatie alvast mee naar huis om te oefenen met het dragen en om te kijken hoe actief je bent voor de operatie. Als je het apparaatje draagt heb je er weinig last van, je kan alles doen wat je normaal ook zou doen. Onze kinderfysiotherapeut zal je helpen met hoe het apparaatje werkt en hoe je het moet gebruiken.
- Voor en na de operatie krijg je een aanvullende afspraak bij de Kinderfysiotherapeut. Tijdens deze afspraak van ongeveer 20 minuten zullen verschillende metingen verricht worden t.a.v. je conditie.
- Voor de operatie wordt het bloedsuikergehalte gecontroleerd. Dit gebeurt door middel van een vingerprik. Dit is een kleine prik waar je weinig last van hebt.
- Bij de nieroperatie krijg je altijd een infuus voor de operatie, je krijgt hier medicijnen door, maar er wordt ook meermaals bloed via afgenomen. We willen hier twee keer één extra buisje bloed uit afnemen, je krijgt dus geen extra prik. We nemen bloed af uit je infuuslijn om te meten hoe je lichaam reageert op het vasten.
- Rondom de operatie krijg je altijd een slangetje in je urinebuis. Daar nemen we een aantal dagen urine uit af voor onderzoek. Ook vragen we aan jou om je urine mee te nemen als je na 4 weken op de polikliniek terugkomt.
- Jij en jouw ouders krijgen 3x een vragenlijst over hoe het met je gaat. De vragenlijst kost je per keer ongeveer 10-15 minuten, de vragenlijst wordt digitaal ingevuld via het KLIK-Portaal van het Prinses Máxima Centrum. Dit doen we om na te gaan hoe belastend het vasten voor jou en je ouders is.
- Er wordt door een patholoog (een arts die gespecialiseerd is in het onderzoeken van lichaamsweefsel en cellen) een stukje afgenomen van je zieke nier tijdens het weefselonderzoek na de operatie, hierin gaan we onderzoeken hoe de cellen hebben gereageerd op het vasten. Hier ondervind je geen last van.

Dingen die we willen weten maar die ook tijdens de standaardbehandeling gemeten worden:

- We gaan je gewicht en lengte dagelijks meten als je in het ziekenhuis bent.
- Alle andere bloedafnames gaan door zoals dat normaal ook gaat.
- We houden de urineproductie bij zoals dat normaal ook gaat.

Over de behandeling

Je wordt altijd de dag voor de operatie al opgenomen in het Prinses Máxima Ziekenhuis. In verband met het onderzoek word je misschien in de vastengroep geloot. Als je in de vastengroep zit die vanaf 18 uur voor de operatie niet meer mag eten, betekent dat dan dat je vanaf 14:00 op de dag voor de operatie niet meer mag eten. Dat is dus vanaf aankomst in het ziekenhuis. Je mag wel water, suikervrije limonade of thee drinken tot vlak voor de operatie, maar er mogen geen voedingstoffen in het drinken zitten.

Zit je in de andere groep, dan mag je tot 6 uur voor de operatie wel eten en drinken, dus tot het slapen gaan. Ook mag je tot vlak voor de operatie nog heldere suikerhoudende dranken drinken.

Risico's

Het (langer) niet eten/vasten heeft in eerdere onderzoeken geen risico's laten zien. We denken dat de kans heel erg klein is dat er vervelende effecten ontstaan als je even niet eet. In zeldzame gevallen kan je een te laag bloedsuikergehalte krijgen. Wij schatten echter in dat de kans hierop klein is, gezien de gekozen vastentijden. Het bloedsuikergehalte zullen we tijdens het vasten zekerheidshalve controleren. Dit kunnen we goed behandelen met een glucose infuus.

Bijwerkingen/Neveneffecten
Het kan gebeuren dat je toch last krijgt van het vasten: door het even niet eten zou je mogelijk een hongergevoel kunnen krijgen of licht draaierig worden. Als je hier last van hebt moeten jij of je ouders dat tegen de verpleegkundige of dokter in het ziekenhuis zeggen. Meestal gaat het vanzelf beter.

Wil je meer weten over wat bijwerkingen zijn? Dan kun je dit vragen aan de arts of de onderzoeksverpleegkundige.

Ongemakken
Voor het onderzoek wordt twee keer een beetje extra bloed afgenomen: namelijk 2 buisjes bloed. Dit is op de dag van opname in het ziekenhuis en vlak voor de operatie. Je hebt zoveel bloed dat je daar niets van merkt. Je hoeft er ook niet extra voor geprikt te worden. Alle andere bloedcontroles gaan zoals normaal.

Tijdens de opname in het ziekenhuis heb je een plasslangetje in verband met de operatie. Hieruit nemen we wat urine af om te onderzoeken. Hier ondervind je geen extra last van. Bij één van de nacontroles willen we nog urine controleren. De urine kan je inleveren in een meegegeven potje. Misschien vind je het spannend om je urine in te leveren, dat is niet erg. Je mag dit altijd tegen je ouders, de dokter of de onderzoeksverpleegkundige zeggen.

De vragenlijsten kunnen soms wat lang duren en het invullen ervan kan misschien saai zijn. Verder hoef je voor het onderzoek geen extra onderzoeken te ondergaan naast de normale behandeling.

Belangrijk om te weten:

- Het is belangrijk dat je je aan het vastendieet houdt voor de operatie tijdens de opname.
- Je mag niet meedoen aan een ander onderzoek zonder dat de onderzoeker dat weet.
- Gebruik je andere medicijnen (of ben je vaak ziek)? Vertel het aan de onderzoeker.
- Je krijgt een kaartje met telefoonnummers en informatie over het onderzoek.
  Wij vragen u om dit kaartje mee te nemen bij elk ziekenhuisbezoek.
- Het is belangrijk dat jij en je ouders de vragenlijsten goed invullen.

Voordelen en nadelen:

Het is belangrijk dat je de mogelijke voor- en nadelen goed afweegt voordat je besluit om mee te doen.

<u>Voordelen:</u>
- Misschien heb je zelf een voordeel van meedoen, namelijk minder schade en een sneller herstel van de nierfunctie na de operatie. We weten niet zeker of dit zal gebeuren.
- Je helpt de onderzoekers om beter te begrijpen hoe een vastendieet kan helpen bij een operatie.
- Je helpt de onderzoekers om in de toekomst de zorg beter te maken voor andere kinderen die ziek worden.

<u>Nadelen:</u>
- Misschien moet je langer vasten voor de operatie (18 uur in plaats van 6 uur).
- Het invullen van de vragenlijsten kost tijd, namelijk 3 keer ongeveer 15 minuten.
- Je moet een aantal dagen thuis een klein apparaatje om je heup dragen.
- Dat je twee extra afspraken krijgt bij de Kinderfysiotherapie.
- Je hebt afspraken waaraan je je zoveel mogelijk moet houden.
- Je zal één keer wat extra urine en 2 buisjes bloed moeten inleveren.
- Misschien vind je het onderzoek & eventjes niet eten spannend.

Vergoeding

Er is geen vergoeding voor meedoen aan het onderzoek.

Jouw rechten

Moet je meedoen?

Nee, je mag **zelf weten** of je meedoet. Als je niet wilt meedoen, dan hoeft dit niet, ook als je ouders dat liever wel willen. Als je wilt meedoen, zet je je handtekening op het formulier. Ook daarna **mag je altijd nog stoppen**, als je liever niet meer wilt. Vertel dat dan wel aan de onderzoeker. Je hoeft niet uit te leggen waarom je stopt.

Als je niet wilt meedoen, dan heeft dat geen invloed op je verdere behandeling als je ziek bent. De behandeling zal doorgaan zoals dat normaal ook zou gaan.

Toestemming intrekken

Als je wilt stoppen, vertel je dit aan de arts of de onderzoeksverpleegkundige. Dit heet: je toestemming intrekken. De informatie die al is verzameld gebruiken we nog voor het onderzoek.

Als je stopt met het onderzoek gaat de behandeling voor de niertumor door zoals dat normaal gaat. Het eventuele vasten zal je niet meer hoeven te doen, de extra vragenlijsten hoef je dan ook niet meer in te vullen.

Jouw gegevens

Voor het onderzoek hebben we drie dingen nodig die van jou zijn:

- ***Persoonsgegevens*** = informatie over wie jij bent, bijvoorbeeld je geboortedatum en waar je woont.

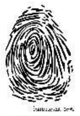

- ***Medische gegevens*** = (ook een soort persoonsgegevens) informatie over je gezondheid, bijvoorbeeld of je ziek bent en of je medicijnen gebruikt.

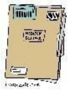

- Je bloed voor bloedonderzoek en stukjes van je ongezonde nier voor onderzoek naar de cellen. Afname hiervan heeft geen (schadelijke) gevolgen voor jou.

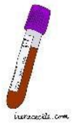

Deze **drie dingen zijn nodig bij het doen van het onderzoek**. Jij en jouw ouders geven toestemming zodat wij deze dingen mogen gebruiken. Wil je meer weten over wat we precies doen met jouw gegevens? Vraag het dan aan je ouders, het staat in hun informatiebrief. Je kunt het ook aan de onderzoeker vragen.

De gegevens (*persoonsgegevens, medische gegevens en bloed & nier*) krijgen **een code,** bijvoorbeeld letters en cijfers. Zo kan een ander niet meteen zien dat de gegevens van jou zijn. Alleen de onderzoeker weet welke code bij wie hoort. Andere mensen zien alleen de code, zij weten dus niet jouw naam.
Ook op de buisjes bloed komt een code en dus niet jouw naam.

Verzekering

Voor iedereen die meedoet aan dit onderzoek is een verzekering afgesloten. De verzekering dekt schade door het onderzoek. Wil je hier meer over weten? Dan kun je dat aan je ouders vragen. In de informatiebrief voor ouders staat het verder uitgelegd. Je kunt het ook aan de arts of onderzoeksverpleegkundige vragen.

Jouw beslissing

Het formulier

Wil je meedoen? Dan zet je een handtekening op het toestemmingsformulier. We hebben ook een handtekening van jouw ouders/voogd nodig. Wanneer iedereen voor deelname getekend heeft, ontvang je een kopie van dit document. Het origineel zullen wij in het ziekenhuis bewaren.

Ook kun je kiezen of we je later mogen vragen voor een vervolgonderzoek. We geven je dan informatie over het nieuwe onderzoek. Dan kun jij opnieuw beslissen of je wilt meedoen.

Meer weten?

Wil je meer weten over onderzoek of over jouw rechten?

Kijk dan op de website van de VKN www.kinderkankernederland.nl of op www.kindenonderzoek.nl

Ondertekening toestemmingsformulier

Als je na zorgvuldige overweging besluit dat je deel gaat nemen aan dit wetenschappelijk onderzoek, dan vragen we je om samen met de dokter het toestemmingsformulier te ondertekenen en er een datum op te zetten. Je krijgt een kopie van deze toestemmingsverklaring na het ondertekenen.

Met vriendelijke groet,

Prof. dr. M.M. van den Heuvel-Eibrink
Kinderarts-oncoloog, hoofdonderzoeker

Prof. dr. M.H.W. Wijnen
Kinderchirurg, hoogleraar kinderoncologische chirurgie

Prof. dr. J.H.J. Hoeijmakers
Moleculair bioloog, moleculair geneticus

Drs. C.A.J. Oudmaijer
Coördinerende arts-onderzoeker

Toestemmingsformulier voor het onderzoek:

- Ik heb de informatie **begrepen**. Ook kon ik vragen stellen. Mijn **vragen** zijn beantwoord.
- Ik had **genoeg tijd om te beslissen** of ik meedoe.
- Ik weet dat ik **niet verplicht** ben om mee te doen.
- Ik begrijp dat ik **altijd mag stoppen** als ik niet meer mee wil doen.

- Ik geef □ **wel** □ **geen** toestemming om mij later te vragen voor een vervolgonderzoek.

Ik wil meedoen aan dit onderzoek.

Naam kind:

Handtekening: Datum :___/___/ __

<u>Dit stuk is voor de arts:</u>

Ik verklaar dat ik het kind volledig heb geïnformeerd over het genoemde onderzoek.

Als er tijdens het onderzoek informatie bekend wordt die de toestemming van het kind zou kunnen beïnvloeden, dan breng ik het kind daarvan tijdig op de hoogte.

Naam arts (of diens vertegenwoordiger):

Handtekening: Datum:___/___/ __

Aanvullende informatie is gegeven door:

Naam:

Functie:

Handtekening: Datum:___/___/ __

Het kind krijgt een volledige informatiebrief mee, samen met een (kopie van de) getekende versie van het toestemmingsformulier.
